# Meroterpenoids Possibly Produced by a Bacterial Endosymbiont of the Tropical Basidiomycete *Echinochaete brachypora*

**DOI:** 10.3390/biom12060755

**Published:** 2022-05-28

**Authors:** Khadija Hassan, Clara Chepkirui, Natalia Andrea Llanos-López, Josphat C. Matasyoh, Cony Decock, Yasmina Marin-Felix, Marc Stadler

**Affiliations:** 1 Department Microbial Drugs, Helmholtz Centre for Infection Research (HZI), German Centre for Infection Research (DZIF), Partner Site Hannover/Braunschweig, Inhoffenstrasse 7, 38124 Braunschweig, Germany; khadija.hassan@helmholtz-hzi.de (K.H.); cchepkirui@biol.ethz.ch (C.C.); natalia.llanos@helmholtz-hzi.de (N.A.L.-L.); 2 Institute of Microbiology, Technische Universität Braunschweig, Spielmannstraße 7, 38106 Braunschweig, Germany; 3 Swiss Federal Institute of Technology in Zurich (ETH), 8092 Zurich, Switzerland; 4 Department of Chemistry, Egerton University, P.O. Box 536, Njoro 20115, Kenya; josphat2001@yahoo.com; 5 Mycothèque de l’Université Catholique de Louvain (BCCM/MUCL), Earth and Life Institute, Microbiology, B-1348 Louvain-la-Neuve, Belgium; cony.decock@uclouvain.be

**Keywords:** endosymbiosis, neomarinone, fumaquinone, endofungal bacteria, *Ralstonia*

## Abstract

A mycelial culture of the African basidiomycete *Echinochaete* cf. *brachypora* was studied for biologically active secondary metabolites, and four compounds were isolated from its crude extract derived from shake flask fermentations, using preparative high-performance liquid chromatography (HPLC). The pure metabolites were identified using extensive nuclear magnetic resonance (NMR) spectroscopy and high-resolution mass spectrometry (HR-MS). Aside from the new metabolites 1-methoxyneomarinone (**1**) and (E)-3-methyl-5-(-12,13,14-trimethylcyclohex-10-en-6-yl)pent-2-enoic acid (**4**), the known metabolites neomarinone (**2**) and fumaquinone (**4**) were obtained. Such compounds had previously only been reported from Actinobacteria but were never isolated from the cultures of a fungus. This observation prompted us to evaluate whether the above metabolites may actually have been produced by an endosymbiontic bacterium that is associated with the basidiomycete. We have indeed been able to characterize bacterial 16S rDNA in the fungal mycelia, and the production of the metabolites stopped when the fungus was sub-cultured on a medium containing antibacterial antibiotics. Therefore, we have found strong evidence that compounds **1**–**4** are not of fungal origin. However, the endofungal bacterium was shown to belong to the genus *Ralstonia*, which has never been reported to produce similar metabolites to **1**–**4**. Moreover, we failed to obtain the bacterial strain in pure culture to provide final proof for its identity. In any case, the current report is the first to document that polyporoid Basidiomycota are associated with endosymbionts and constitutes the first report on secondary metabolites from the genus *Echinochaete*.

## 1. Introduction

Basidiomycota are known for their capacity to produce unique natural products with manifold therapeutic and economic benefits, and in particular their tropical species are still widely underexplored [1]. In our continuous study of tropical basidiomycetes from Kenya, we have already reported several new secondary metabolites from rare and new species [2,3,4,5]. The current study deals with the genus *Echinochaete*, whose species are mainly distributed in tropical to subtropical areas of Africa, America and Asia. The genus was described for the first time by Reid in 1963, belongs to the family Polyporaceae [6], and currently contains six species including *Echinochaete maximipora, E. brachypora, E. cinnamomeosquamulosa, E. ruficeps, E. megalopora*, and *E. russice* [7].

Members of this genus are characterized by having basidiocarps with short, lateral stipes, a dimitic hyphal system with dextrinoid arboriform skeleto-binding hyphae and clamped generative hyphae, spinulose setoid elements on the pileus surface and in the hymenium, and cylindrical basidiospores [6,7].

No secondary metabolites have so far been reported from *Echinochaete*. An extract of the strain *Echinochaete brachypora* (MUCL 56080) showed moderate antimicrobial activities in a preliminary screening and was thus selected for identification of its active principles. Below, we report the isolation and identification of its secondary metabolites, as well as a preliminary study on their potential origin.

## 2. Materials and Methods

### 2.1. Fungal Materials

The strain MUCL 56080 was isolated from a specimen collected by C. D. and J. C. M. in Mount Elgon National Reserve (1°7′6″ N, 34°31′30″ E) in Kenya in April 2016. A dried specimen and corresponding culture were deposited at the Mycothèque de l’Université Catholique de Louvain (MUCL, Belgium). The species was identified by morphological studies and comparison of the internal transcribed spacer (ITS)-nrDNA sequences with others from Basidiomycota deposited in GenBank and the in-house collection at MUCL.

### 2.2. Cultivation of the Fungal Strain

*Echinochaete* cf. *brachypora* MUCL 56080 was cultivated in Q6 1/2 medium (10 g/L glycerol, 2.5 g/L d-glucose, 5 g/L cotton seed flour; pH 7.2). Mycelia from a well-grown culture on YM (yeast-malt) agar plate (10 g/mL malt extract, 4 g/mL yeast extract, 4 g/mL D-glucose supplemented with 1.5% agar, pH = 6.3) were cut into small plugs using a cork borer (7 mm), and 5 pieces were inoculated in a 500 mL Erlenmeyer flask containing 200 mL of medium. The cultures were incubated at 24 °C on a rotary shaker (140 rpm). The growth of the fungus was monitored by checking the amount of free glucose with Medi-test glucose (Macherey-Nagel, Düren, Germany). The fermentation was terminated 2 days after glucose depletion.

In a control experiment, the strain was passed through a YM agar plate containing 50 mg/L of penicillin G and streptomycin sulfate and the fermentation was repeated afterwards. For scale-up cultivation, 5 L of Q6 medium was prepared in a 500 mL Erlenmeyer flask containing 200 mL of media. The fungal cultures (which were initially passed through media supplemented with antibiotics and then re-sub-cultured in YM media without antibiotics) were cut into small plugs using a cork borer (7 mm), and 5 pieces were inoculated in each flask. The cultures were incubated at 24 °C on a rotary shaker (140 rpm). The growth of the fungus was monitored by checking the amount of free glucose with Medi-test glucose (Macherey-Nagel). The fermentation was terminated 2 days after glucose depletion. However, the compounds **1**–**4** treated below were not detected anymore and the production could not be reinstated.

### 2.3. Preparation of the Extracts

Supernatant and mycelia from the cultures were separated by filtration. The supernatant was extracted with an equal amount of ethyl acetate and filtered through anhydrous sodium sulfate. The resulting ethyl acetate extract was evaporated to dryness by a rotary evaporator, resulting in 37 mg crude product. The mycelia were extracted with 200 mL of acetone in an ultrasonic bath for 30 min and filtered, and the collected filtrate was evaporated. The remaining aqueous phase was suspended in an equal amount of distilled water and extracted with an equal amount of ethyl acetate. Ethyl acetate extract was evaporated to dryness, yielding 58 mg crude product.

### 2.4. General Experimental Procedures

NMR spectra (H, _13_C, COSY, HSQC, HMBC, ROESY) were recorded with a Bruker Daltonics (Bremen, Germany) Avance III 700 spectrometer with a 5 mm TXI cryoprobe (1H 700 MHz, 13C 175 MHz, 15N 71 MHz) and Bruker AV III-600 (1H 500 MHz, 13C 150 MHz) spectrometers (sample temperature: 298 K). HR-ESIMS (high-resolution electrospray ionization mass spectrometry) spectra were recorded after purification with an Agilent 1200 infinity series HPLC-UV system (Agilent Technologies, Santa Clara, CA, USA) (column size: 2.1 mm⋅50 mm, packing: 1.7 µm, (Waters) Acquity UPLC BEH C18 solvent A: H_2_O + 0.1% formic acid, solvent B: acetonitrile + 0.1% formic acid, elution gradient: 5% solvent B for 0.5 min, increasing solvent B to 100% within 19.5 min, 100% solvent B for 5 min, flow rate: 0.6 mL/min, UV-vis detection at λ = 200–600 nm) and ESI-TOF-MS analysis (maXis™ system, Bruker,; scan range: 100–2500 m/z, capillary voltage: 4500 V, drying temperature: 200 °C). The NMR and mass spectra for all compounds are deposited in the Supplementary information (Figures S1–S26).

### 2.5. Isolation of Compounds from Strain MUCL 56080

The mycelial and supernatant crude extracts were combined because of the similar chemical profile of the two extracts. The combined crude extract was dissolved in methanol and filtered through a Strata™-X 33 μm Polymeric Reversed Phase Tube (Phenomenex, Aschaffenburg, Germany). For a further separation of the compounds, the crude extracts were fractionated with a preparative HPLC Gilson GX270 Series HPLC system (Gilson Inc., Middleton, WI, USA) equipped with VP preparative (Kromasil) 250 mm × 20 mm, 7 μm C18 column (Macherey-Nagel). The extract (95 mg) was dissolved in methanol and subjected to preparative reverse phase HPLC using a mobile phase consisting of deionized water with 0.05% of trifluoroacetic acid (solvent A) and acetonitrile with 0.05% of trifluoroacetic acid (solvent B) with a flow rate of 15 mL/min. A gradient from 40% to 100% acetonitrile for 60 min, followed by isocratic elution at 100% solvent B for 5 min, was applied. UV detection was carried out at 210, 254, and 350 nm. A total of 12 fractions (F1–F12) were collected based on the observed peaks (two fractions were collected per minute)

Fractions F-2 and F3 were further purified by reverse phase HPLC (solvent A/solvent B), elution gradient 68–80% solvent B for 20 min, followed by a gradient shift from 68 to 100% in 5 min, and finally isocratic condition at 100% solvent B for 5 min to give 0.78 mg of compounds **3** and 0.7 mg of **4,** respectively. A preparative Kromasil C18 HPLC column (5 µm; 250 mm × 4.6 mm) was used as stationary phase. The same column was also used to purify the other compounds. F6 was purified to obtain compound **1** (0.5 mg) with an elution gradient 80% B for 3 min followed by gradient increase to 95% B in 20 min. Fraction F-4 was purified with elution gradient of 75–85% solvent B for 20 min, followed by a gradient shift from 85 to 100% in 5 min, and finally isocratic condition at 100% solvent B for 5 min to afford 0.7 mg of compound **4**.

### 2.6. DNA Extraction and PCR

Genomic DNA was extracted from a 1-week-old mycelial culture on YM agar plate following the Fungal gDNA Miniprep Kit EZ-10 Spin Column kit protocol (NBS Biologicals, Cambridgeshire, UK). The polymerase chain reaction (PCR) was performed to amplify partial sequences of DNA regions, i.e., the internal transcribed spacer region (ITS) using the standard primers ITS1F [8] and ITS4 [9], the 28S large subunit (LSU) using the primers LR0R and LR07 [10], and the 16S ribosomal RNA with the primers F27 (5′-AGAGTTTGATCMTGGCTCAG-3′) and R1492 (5′-TACGGYTACCTTGTTACGACTT-3′) [11]. PCR products were checked by electrophoresis using 1.5% agarose gel and 1X Tris-acetate-EDTA buffer (TAE) and sequenced using Sanger Cycle Sequencing method at Microsynth Seqlab GmbH (Göttingen, Germany). Primers used for sequencing were the same used for the PCR amplification, except for 16S rRNA, for which the additional primer R518 (5′-CGTATTACCGCGGCTGCTGG-3′) was also used [12]. To verify the presence of the bacterium, the extraction of genomic DNA and the amplification of 16S rRNA was repeated 3 times with different DNA extraction kits.

Consensus sequences were obtained employing the Geneious^®^ 7.1.9 program [13]. The sequences were then compared to reference data available by using the Basic Local Alignment Search Tool (BLAST, https://blast.ncbi.nlm.nih.gov/Blast.cgi (accessed on 8 March 2022)).

### 2.7. Isolation Attempts of Fungal Endobacteria

The strain *E. brachypora* MUCL 56080, well grown on YM medium (pH 6.3), was sub-cultured using the streaking plate method on plates with the medium 5336 [14] supplemented with the antifungal cycloheximide (100 ppm), in order to isolate bacterial strains from the fungal cultures. The plates were maintained at 30 °C for 30 days.

### 2.8. Antimicrobial Assay

The Minimal Inhibitory Concentrations (MICs) of the compounds **1**–**4** were determined in a serial dilution assay as described previously by [15,16,17] and were carried out in 96-well microtiter plates. The test organisms were the Gram-positive bacterium *Bacillus subtilis* (DSM 10), the Gram-negative bacterium *Escherichia coli* (DSM 498), and the filamentous fungus *Mucor plumbeus* (MUCL 49355). A detailed protocol can be found in the Supplementary Materials.

## 3. Results

### 3.1. Structure Elucidation

**Figure 1:**
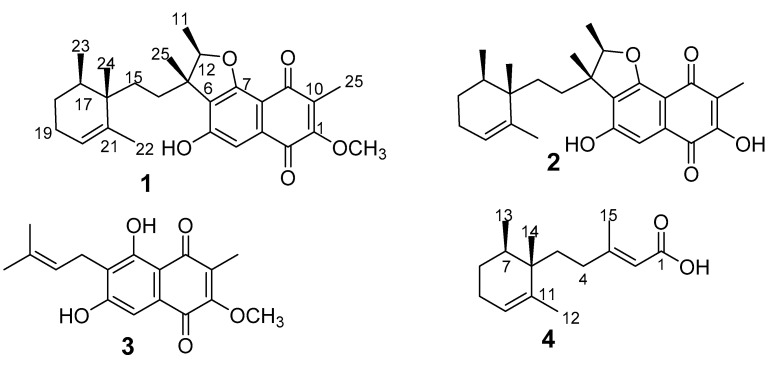
Chemical structures of compounds **1**–**4** isolated from MUCL 56080.

The crude extracts exhibited activity against *B. subtilis* in the initial screening and therefore were selected for further studies. Bioassay-guided fractionation allowed the identification of the active metabolites. To obtain more extract, scale-up fermentation was carried out. However, the active principles were not detected again in our extracts. Therefore, we went back to the small-scale extracts that we used in the bioassay-guided fractionation. Four compounds (**1**–**4**) were purified from the active fractions by preparative HPLC (Figure 1).

Compound **1** (1-methoxy neomarinone), with the molecular formula C_27_H_34_O_5_ established from the HR-MS data, was isolated from both the supernatant and the mycelial extracts. Two methyl doublets with resonance at δ 0.86 (H_3_-23) and H_3_-11 (δ 1.38) and an additional five methyl singlets at δ at 0.82 (H_3_-24), 1.31 (H_3_-25), 1.59 (H_3_-22), and H-1.96 (H_3_-26) were recorded in the ^1^H NMR spectrum (also compare Table 1). A total of 27 carbons were identified from the 13C which were further classified as 7 methyl, 4 methylene, 4 methine groups, and 12 non-protonated carbons based on the Distortionless Enhancement by Polarization Transfer (DEPT) data. Three spin systems were established from the COSY (correlation spectroscopy) data. The first spin system was deduced from the COSY correlations of H-17 to H_3_-23/H_2_-18 and H_2_-19 to H_2_-18/H-20. The structure assignment of the trimethylcyclohexene moiety was facilitated by the HMBC (heteronuclear multiple bond correlation) correlations of the three methyl groups. Cross peaks in the HMBC spectrum were recorded between H_3_-22 to C16/C-20/C21, H_3_-23 to C16/C-17/C18, and H-_3_ 24 to C16/C17. The naphthalene-1,4-dione moiety connected to the furan ring elucidation was facilitated by the HMBC correlations of H_3_-25 to C1/C9/C10, H_3_-11 to C12/C13, H_3_-25 to C6/C12/C13/C14, and H-4 to C-2/C-3/C5/C6/C7. The furan ring was established from the HMBC cross peak of H-12 to C7 and the down field shift of C12 (δ 88.4) and C7 (δ 161.7). The typical ^13^C signals for the naphthalene-1,4-dione moiety with resonance at δ 181.4 (C2) and δ 183.5 (C9) completed the elucidation of this moiety. The hydroxy group and the methoxy group were attached to C5 and C1, respectively, based on the chemical shift of C5 (δ 158.7) and the HMBC correlations of methoxy group protons to C1 (δ 157.6). The two moieties were connected based on the COSY correlations of H2-14 to H2-15 and the HMBC correlations of the methyl groups’ protons H_3_-25 to C14 and H_3_-24 to C15, and consequently concluding the structure of **1**.

Compound **1** features a similar structure as the meroterpenoid neomarinone (**2**) with the difference being the methoxy group in **1**. Neomarinone was first reported by Hardt and co-workers [18] from the marine filamentous actinomycete strain CNH-099. The initial structure reported featured a cyclopentane ring, rather than the sesquiterpene. The structure was later corrected after feeding experiments and new NMR data indicated the cyclopentane ring, and hence the structure of **2** was concluded to be the correct structure of neomarinone [19]. The ROESY (rotating frame Overhauser enhancement spectroscopy) data of **1** revealed a cross peak between H_3_-24 and H_3_-11/H_3_-24 and between H_3_-23 and H_3_-25. Similar correlations were reported for neomarinone (**2**) [19], and therefore a similar stereochemistry was assigned to the new derivative **1**. Compound **3** was identified as the prenylated naphthoquinone fumaquinone, previously reported from *Streptomyces fumanus*, by comparison of the NMR data to those reported in the literature [20].

Compound **4**, which was isolated from both the supernatant and the mycelia extracts, had the molecular formula C_15_H_24_O_2_ deduced from the HR-MS data. This compound shares similar a trimethylcyclohexene moiety as in **1** with a side chain. The HMBC correlations of H-2 to C1/C4/C15, H_3_-15 to C2/C3/C4, along with the COSY correlations of H_2_-4/H_2_-5 confirmed the structure of **4** as 3-methylpent-2-enoic acid. The carboxylic acid moiety was concluded from the chemical shift of C1 (δ 167.8) and the two oxygen atoms predicted from the molecular formula. The side chain was connected to the cyclohexene moiety through C5 because of the HMBC correlations of H_3_-14 to C5. Therefore, the structure of **4** was unambiguously assigned as (*E*)-3-methyl-5-(-12,13,14-trimethylcyclohex-10-en-6-yl)pent-2-enoic acid.

### 3.2. Physicochemical Data for Compounds ***1***–***4***

Compound **1**: Brown solid. UV (MeOH): 224 nm, 267 nm, 300 nm; ESIMS *m*/*z* 899.5403 [2M + Na]^+^, 461.2500 [M + Na]^+^, 439.2575 [M + H]^+^, HRESIMS *m*/*z* 439.2483 [M + H]^+^ calcd for C_27_H_35_O_5_, 439.2484.

Compound **2**: Brown solid. UV (MeOH): 222 nm, 265 nm, 315 nm; ESIMS *m*/*z* 871.5093 [2M + Na]^+^, 449.22 [M + Na]^+^, 425.2265 [M + H]^+^, HRESIMS *m*/*z* 425.2326 [M + H]^+^ calcd for C_26_H_33_O_5_, 425.2328.

Compound **3**: Yellow solid. UV (MeOH): 222 nm, 269 nm, 306 nm; ESIMS *m*/*z* 627.1250 [2M + Na]^+^, 325.1250 [M + Na]^+^, 303.0696 [M + H]^+^, HRESIMS *m*/*z* 303.1227 [M + H]^+^ calcd for C_17_H_19_O_5_, 303.1232; found, 475.3059.

Compound **4**: Yellow oil. UV (MeOH): 224 nm; ESIMS *m*/*z*, 259.1158 [M + Na]^+^, 237.1105 [M + H]^+^, HRESIMS *m*/*z* 237.1847 [M + H]^+^ calcd for C_15_H_25_O_2_, 237.1854.

### 3.3. Molecular Identification of MUCL 56080

Blast search results revealed that MUCL 56080 had a 98.60% (ITS) and 99.08% (LSU) nucleotide similarity to *E. brachypora* and *Echinochaete* sp., respectively.

A phylogeny of *Echinochaete,* based on LSU and ITS sequences, showed that *E. brachypora* as commonly accepted [21] is a complex of four species: *E. brachypora* s.s., from the Neotropics, *Echinochaete* sp., from SE Asia, and two species from tropical Africa, with a distribution over high land (or eastern Africa) vs. low land (or central Africa). MUCL 56080 collected from Mt. Elgon belongs to the African high land lineage within the *E. brachypora* lineage s.l. (Figure S27).

### 3.4. Identification and Isolation of Endofungal Bacteria

A literature search of the isolated compounds neomarinone (**2**) and fumaquinone (**3**) indicated that the isolated metabolites have only been reported before from bacteria and more specifically *Streptomyces* species [18,20]. Therefore, the question whether the metabolites were actually produced by the fungus or by an endophytic bacterium arose. To confirm this hypothesis, the genomic DNA extracted from the strain MUCL 56080 was subjected to the amplification of the 16S region, which is considered the barcode locus for bacterial identification [22]. The amplification of the 16S rRNA locus was successful for the DNA sample derived from the strain, corroborating the presence of a bacterium associated with our strain. To verify the presence of the bacterium, the extraction of genomic DNA and the amplification of 16S rRNA was repeated three times with different DNA extraction kits.

**Figure 2:**
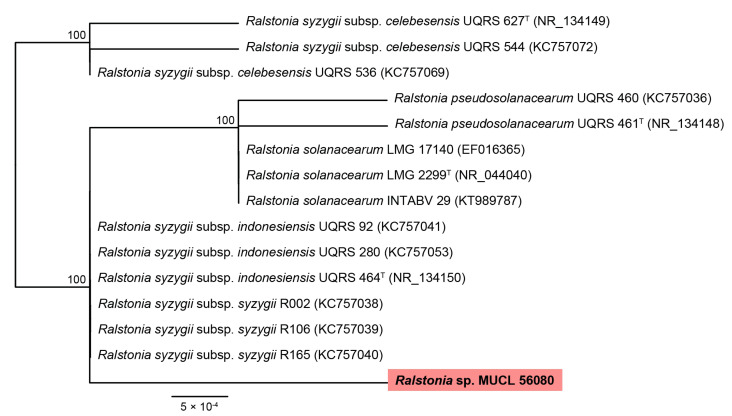
RAxML phylogram obtained from 16S sequences of taxa included in *Ralstonia solanacearum* species complex. Bootstrap support values ≥ 70 are indicated along branches. Branch lengths are proportional to distance. Ex-type strains are indicated with ^T^. The maximum-likelihood (ML) analyses employed RAxML on the CIPRES portal (www.phylo.org, accessed on 12 May 2022) using RAxML-HPC BlackBox v8.2.12 with default parameters. Our strain studied is highlighted using pink colour.

According to blast search results and a phylogenetic study based on the 16S rRNA sequences, the bacterium present in the *E. brachyphora* strain belongs to the *R. solanacearum* species complex (Figure 2). The bacterial 16S rRNA detected in MUCL 56080 had a nucleotide similarity of 99.87% to *R. syzygii*. Unfortunately, it will not be possible to reach a more conclusive identification without the availability of an axenic culture.

**Figure 3:**
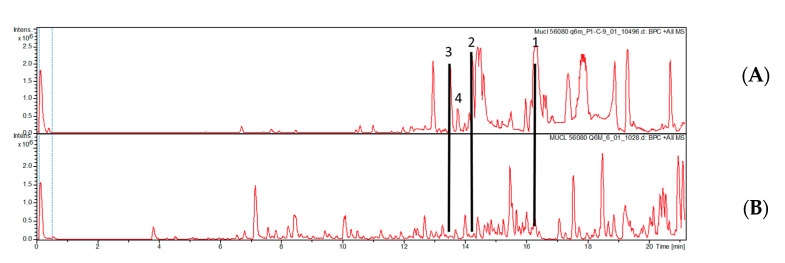
HPLC chromatogram (210 nm) of the crude extracts of *E. brachypora* MUCL 56080 grown in Q61/2 media (**A**) and Q6 + antibiotics (**B**). Compounds **1**–**4** were only produced in the absence of the antibacterial antibiotic.

No bacterial growth was observed on the plates supplemented with antifungal agents, thus impeding a concise identification of the endofungal symbiont. There is also only indirect evidence that the bacterium actually produced the metabolites isolated from the *Echinochaete* strain. However, as shown in Figure 3, the production of compounds **1**–**4** ceased when the strain was transferred to culture media supplemented with antibacterial antibiotics.

### 3.5. Biological Activity

The compounds **1**–**4** were evaluated for antimicrobial activity against *Bacillus subtilis* DSM 10, *Escherichia coli DSM 498*, and *Mucor plumbeus* MUCL 49355 (Table 2). Compounds **1**–**3** were active against *B. subtilis* with an MIC of 4.6 µg/mL, while **4** was devoid of significant activities. The antimicrobial activity of neomarinone has not been reported before. Neomarinone was reported to be moderately cytotoxic towards HCT-116 colon carcinoma and the 60 cancer cell line panel of NCI [18]. Similar compounds such as the cytotoxic and antibacterial marfuraquinocins have since been reported from *Streptomyces niveus* (SCSIO 3406) collected in the deep South China Sea [23]

## 4. Discussion

After structure elucidation, the compounds isolated were identified as neomarinone (**2**) and its derivatives, which are only known from bacteria. Even an extensive literature search did not result in a single report of such metabolites from fungi. This brought back the question as to why we could not detect the compounds in our scale-up fermentations after they had been detected in our small-scale screening. The only difference between our small-scale cultivation and scale-up fermentation was that the cultures used for large-scale fermentation had been passed through YM medium containing antibiotics. Hence, we speculated that perhaps the metabolites were produced by a bacterium in our fungal culture, which was then lost when the strain was cultivated in media supplemented with antibiotics during sub-culturing.

To reaffirm our suspicions, we revived the strain from the MUCL holdings (where it had been deposited without any contact with antibacterial antibiotics) and cultured it in parallel with and without the presence of antibacterial agents. Interestingly, the compounds could be detected only in the cultures cultivated without antibiotics (Figure 2). In nature, there are cases in which some properties of fungi come from their endosymbiont bacteria, as occurs with the association between the phytopathogenic zygomycete *Rhizopus microsporus* and the bacterium *Paraburkholderia rhizoxinica*. A major virulence factor of *R*. *microsporus* is the toxin rhizoxin, synthesized by the bacterium within it [24].

We also attempted to isolate the bacteria from the culture, but we were not successful. There are several reports in the literature where other researchers have also failed to culture endosymbiontic bacteria outside their host cells with ordinary culture media. One example is the *Holospora*, an endonuclear symbiont of the genus *Paramecium* [25]. Moreover, according to Schüßler et al. [26], most known intrafungal bacteria cannot be cultured by conventional methods. On the other hand, there are also some reports where cultivation of fungal endobacteria from their hosts was successful. For instance, the bacterial symbionts of the glomeromycete *Gigaspora margarita* could be cultured [27], and the endobacterium RrF4 was isolated from crushed mycelium of *Serendipita indica* (formerly known as *Piriformospora indica*) [28].

We then decided to take the mycelia and amplified the 16S loci in which we obtained a hit, and the bacterium was identified as *Ralstonia* sp. We further performed the same procedure for other *Echinochaete* sp. strains, and they all had similarities to the *Ralstonia* sp. complex (Supplementary Materials).

The genus *Ralstonia* is a member of the β-proteobacteria group, and contains species that can be opportunistic human pathogens including *R. pickettii, R. insidiosa*, and *R. mannitolilytica* [29], as well as devastating plant pathogens such as *R. solanacearum* [30]. Others strains of *R. pickettii* have been shown to play an important role in ecosystems as degraders of xenobiotic and recalcitrant compounds [31,32]. The species of *Ralstonia* can be found in a wide variety of environments. In particular, *R. pickettii* and *R. mannitolilytica* are frequently observed in aquatic habitats, while *R. solanacearum* is commonly referred to as soil-borne bacterium [33,34]. Bacteria that inhabit fungi intracellularly, or endosymbiotically, have been described for more than three decades [35,36,37,38,39] and have been documented from all parts of the world [39,40]. Bacteria associated endosymbiotically with fungal cells have been predominantly identified as belonging to the β-proteobacteria group, and were first described as Bacteria-Like Organisms (BLOs) in 1970s [41]. Members of the aforementioned group (including the genera *Burkholderia* and *Pandoraea*) are known to be of beneficial significance in fungal–bacterial interactions [24,38]. The bacteria have diverse functions, including promoting hyphal growth, mycorrhiza formation, and production of secondary metabolites [42,43,44,45,46,47]. Mutually beneficial relationships of fungi and bacteria have also been observed between the phytopathogenic *Ralstonia solanacearum* and different fungal species across diverse taxa, including basidiomycetes. The bacterium produces the secondary metabolite ‘ralsolamycin’ using a non-ribosomal peptide synthetase–polyketide synthase hybrid, which contributes to bacterial invasion into the fungal hyphae and induces chlamydospore formation [48].

The fact that the region 16S rRNA of *Ralstonia* sp. was identified in the studied fungal strains is an important advancement and could explain the production of compounds **1** and **2**, supporting one of the statements raised previously that suggest an endosymbiotic relationship between the fungal isolates and a bacterium. It is pertinent to mention that compounds **1** and **2** are only known from a marine Actinobacterium and a bacterium isolated from soil, respectively. Although some *Ralstonia* species live in soil, they have to our knowledge not yet been reported from marine environments.

However, the fact that the compounds that we found in the fungal culture that still contained the living bacteria are all known from Actinobacteria such as *Streptomyces*, rather than *Ralstonia,* should give rise to some doubt. Actually, we cannot exclude that a *Streptomyces* was present in the fungus in addition to the *Ralstonia* species, even though we can expect that the employed PCR technique would also work well for detection of a *Streptomyces*, if any were present in the fungus. Even though *Streptomyces* species do not usually occur as endofungal symbionts but are typically soil inhabiting saprotrophs, it cannot be excluded that there are hitherto undiscovered lineages of this versatile organism group that still remain to be discovered. On the other hand, it is well known that the same compound class can be present in different phylogenetically unrelated organisms, and the genus *Ralstonia* has not yet been studied as exhaustively for secondary metabolites as has *Streptomyces*. Additional attempts to culture the *Ralstonia* endosymbionts using different culture media and experimental parameters should be carried out in the future in an attempt to accomplish this task and finally assure that the *Ralstonia* spp. are indeed the source of the meroterpenoids.

## 5. Conclusions

In conclusion, the present study reports on the isolation of four secondary metabolites from the hitherto unexplored African basidiomycete *Echinochaete brachyphora*, of which two are new. Furthermore, the molecular study based on 16S rRNA suggested the presence of endofungal bacterium belonging to the *R. solanocearum* species complex in a polyporoid basidiomycete for the first time. More extensive experiments, including a variation in culture conditions, should be carried out in an attempt to isolate the *Ralstonia* sp. and/or other bacteria that are present in the fungal mycelium. However, this is beyond the scope of the current study.

Even though we were unable to obtain the bacterial endosymbionts in axenic culture, our preliminary results should give impetus to study more of these relationships between the polypores and their associated bacteria.

## Figures and Tables

**Table 1:** NMR data for compound **1** and **4** (^1^H 700 MHz, ^13^C 175 MHz, in acetone-d_6_) and **2** (^1^H 500 MHz, ^13^C 125 MHz, in acetone-d_6_).

	1		2		4	
No.	^13^C/HSQC	^1^H	^13^C/HSQC	^1^H	^13^C/HSQC	^1^H
1.	157.6, C		153.7, C		167.8, C	
2.	181.4, C		181.5, C		115.9, CH	5.69 (q), *J* = 1.1Hz
3.	134.7, C		132.7, C		161.7, C	
4.	108.9, CH	7.14 (s)	108.6, CH	1.17 (s)	36.4, CH_2_	1.81 (m), 2.13 (m)
5.	158.7, C		158.2, C		35.4, CH_2_	1.55 (m), 1.61 (m)
6.	128.1, C		129.1, C		41.3, C	
7.	161.7, C		161.8, C		34.2, CH	1.75 (m)
8.	109.8, C		109.9, C		27.8, CH_2_	1.47 (m)
9.	183.5, C		183.5, C		26.2, CH_2_	1.92 (m), 1.98 (m)
10.	133.3, C		121.3, C		125.4, CH	5.45 (m)
11.	16.2, CH_3_	1.38 (d) *J* = 6.7 Hz	16.2, CH_3_	1.37 (d) *J* = 6.5 Hz	139.9, C	
12.	88.4, CH	4.76 (q) *J* = 6.6 Hz	88.2, CH	4.76 (q) *J* = 6.6 Hz	19.4, CH_3_	1.63 (s)
13.	47.4, C		47.5, C		16.2, CH_3_	0.89, (m)
14.	33.2, CH_2_	1.66 (m)	33.1, CH_2_	1.67 (m)	21.3, CH_3_	0.90, (s)
15.	32.2, CH_2_	1.29 (m), 1.46 (m)	32.1, CH_2_	1.30 (m), 1.46 (m)	18.9, CH_3_	2.16 (d), *J* = 1.2 Hz
16.	40.9, C		40.9, C			
17.	34.1, CH	1.79 (m)	34.1, CH	1.78 (m)		
18.	27.8, CH_2_	1.39 (m), 1.46 (m)	27.8, CH_2_	1.43 (m)		
19.	26.0, CH_2_	1.88 (m), 1.93 (m)	26.0, CH_2_	1.9 0 (m)		
20.	124.4, CH	5.36 (m)	124.6, CH	5.36 (m)		
21.	140.4, C		140.4, C			
22.	19.4, CH_3_	1.59 (s)	19.4, CH_3_	1.58 (s)		
23.	16.2, CH_3_	0.86 (d) *J* = 7.1 Hz	16.2, CH_3_	0.86 (d) *J* = 6.9 Hz		
24.	21.7, CH_3_	0.82 (s)	21.7, CH_3_	0.82 (s)		
25.	20.2, CH_3_	1.31 (s)	20.1, CH_3_	1.31 (s)		
26.	9.4, CH_3_	1.96 (s)	8.7, CH_3_	1.95 (s)		
OCH_3_	60.8, CH_3_	3.98 (s)				

**Table 2:** Antimicrobial activities of compounds **1**–**4.**

	MIC (μg/mL)				
Test Organism	1	2	3	4	Positive Control
*Bacillus subtilis* DSM10	4.6	4.6	4.6	-	2.3 ^a^
*Escherichia coli* DSM 498	-	-	-	-	2.3 ^a^
*Mucor plumbeus* MUCL 49355	-	-	-	-	9.4 ^b^

^a^ Ciprofloxacin, ^b^ nystatin—no activity.

## Data Availability

Not applicable.

## Supplementary Materials

The following are available online at https://www.mdpi.com/article/10.3390/biom12060755/s1. Figure S1: ESIMS data for compound 1; Figure S2: ^1^H NMR spectrum (acetone-d4, 500 MHz) of (1); Figure S3: ^13^C NMR spectrum (acetone-d4, 700 MHz) of (1); Figure S4: HSQC spectrum in acetone-d4 (^1^H 500 MHz, ^13^C 125) of (1);Figure S5: HMBC spectrum in acetone-d6 (^1^H 500 MHz, ^13^C 125 MHz) of (1); Figure S6: COSY spectrum in acetone-d6 (^1^H 500 MHz) of (1); Figure S7: ROESY spectrum in acetone-d6 (^1^H 500 MHz) of (1); Figure S8: ESIMS data for compound 2; Figure S9: ^1^H NMR spectrum (acetone-d4, 500 MHz) of (2) Figure S10: ^13^C NMR spectrum (acetone-d4, 700 MHz) of (2); Figure S11: HSQC spectrum in acetone-d4 (1H 500 MHz, 13C 125) of (2); Figure S12: HMBC spectrum in acetone-d6 (1H 500 MHz, 13C 125 MHz) of (2); Figure S13: ROESY spectrum in acetone-d6 (1H 500 MHz) of (2); Figure S14: COSY spectrum in acetone-d6 (1H 500 MHz) of (2); Figure S15: ESIMS data for compound 3; Figure S16: 1H NMR spectrum (acetone-d4, 500 MHz) of (3); Figure S17: ^13^C NMR spectrum (acetone-d4, 700 MHz) of (3); Figure S18: HSQC spectrum in acetone-d4 (1H 500 MHz, ^13^C 125) of (3); Figure S19: HMBC spectrum in acetone-d6 (^1^H 500 MHz, ^13^C 125 MHz) of (3); Figure S20: COSY spectrum in acetone-d6 (^1^H 500 MHz) of (3); Figure S21: ESIMS data for compound 4; Figure S22: 1H NMR spectrum (acetone-d4, 500 MHz) of (4); Figure S23: ^13^C spectrum (acetone-d4, ^13^C 125) of (4); Figure S24: HSQC spectrum in acetone-d4 (1H 500 MHz, ^13^C 125) of (4); Figure S25: HMBC spectrum in acetone-d6 (^1^H 500 MHz, ^13^C 125 MHz) of (4); Figure S26: ROESY spectrum in acetone-d6 (^1^H 500 MHz) of (4); Figure S27: Phylogeny of *Echinochaete*, based on LSU and ITS

## References

[1] Sandargo B., Chepkirui C., Cheng T., Chaverra-Muñoz L., Thongbai B., Stadler M., Hüttel S. (2019). Biological and chemical diversity go hand in hand: Basidiomycota as source of new pharmaceuticals and agrochemicals. Biotechnol. Adv..

[2] Cheng T., Chepkirui C., Decock C., Matasyoh J.C., Stadler M. (2019). Sesquiterpenes from an Eastern African medicinal mushroom belonging to the genus *Sanghuangporus*. J. Nat. Prod..

[3] Chepkirui C., Cheng T., Sum W.C., Matasyoh J.C., Decock C., Praditya D.F., Wittstein K., Steinmann E., Stadler M. (2019). Skeletocutins A-L: Antibacterial agents from the Kenyan wood-inhabiting basidiomycete, *Skeletocutis* sp.. J. Agric. Food Chem..

[4] Cheng T., Chepkirui C., Decock C., Matasyoh J.C., Stadler M. (2019). Skeletocutins M–Q: Biologically active compounds from the fruiting bodies of the basidiomycete *Skeletocutis* sp. collected in Africa. Beilstein J. Org. Chem..

[5] Chepkirui C., Matasyoh J.C., Decock C., Stadler M. (2017). Two cytotoxic triterpenes from cultures of a Kenyan *Laetiporus* sp. (Basidiomycota). Phytochem. Lett..

[6] Reid D.A. (1963). New or interesting records of Australasian Basidiomycetes: V. Kew Bull..

[7] Sotome K., Hattori T., Ota Y., Lee S.S., Vikineswary S., Abdullah N., Kakishima M. (2009). Taxonomic study of Asian species of *Echinochaete* (Polyporaceae, Basidiomycota) and description of *E. maximipora* sp. nov. Mycol. Prog..

[8] Gardes M., Bruns T.D. (1993). ITS primers with enhanced specificity for basidiomycetes-application to the identification of mycorrhizae and rusts. Mol. Ecol..

[9] White T.J., Bruns T., Lee S., Taylor J., Innis M.A., Gelfand D.H., Sninsky J.J., White T.J. (1990). Amplification and direct sequencing of fungal ribosomal RNA genes for phylogenetics. PCR Protocols: A Guide to Methods and Applications.

[10] Vilgalys R., Hester M. (1990). Rapid genetic identification and mapping of enzymatically amplified ribosomal DNA from several *Cryptococcus* species. J. Bacteriol..

[11] Eden P.A., Schmidt T.M., Blakemore R.P., Pace N.R. (1991). Phylogenetic analysis of *Aquaspirillum magnetotacticum* using polymerase chain reaction-amplified 16s rRNA-specific DNA. Int. Union Microbiol. Soc..

[12] Muyzer G., De Waal E.C., Uitterlinden A.G. (1993). Profiling of complex microbial populations by denaturing gradient gel electrophoresis analysis of polymerase chain reaction-amplified genes coding for 16S rRNA. Appl. Environ. Microbiol..

[13] Kearse M., Moir R., Wilson A., Stones-Havas S., Cheung M., Sturrock S., Buxton S., Cooper A., Markowitz S., Duran C. (2012). Geneious Basic: An integrated and extendable desktop software platform for the organization and analysis of sequence data. Bioinformatics.

[14] Khosravi Babadi Z., Ebrahimipour G., Wink J., Narmani A., Risdian C. (2021). Isolation and identification of *Streptomyces* sp. Act4Zk, a good producer of staurosporine and some derivatives. Lett. Appl. Microbiol..

[15] Becker K., Wessel A.C., Luangsa-Ard J.J., Stadler M. (2020). Viridistratins A-C, antimicrobial and cytotoxic benzo fluoranthenes from stromata of *Annulohypoxylon viridistratum* (Hypoxylaceae, Ascomycota). Biomolecules.

[16] Kemkuignou B.M., Treiber L., Zeng H., Schrey H., Schobert R., Stadler M. (2020). Macrooxazoles a–d, new 2,5-disubstituted oxazole-4-carboxylic acid derivatives from the plant pathogenic fungus *Phoma macrostoma*. Molecules.

[17] Hassan K., Kemkuignou B.M., Stadler M. (2021). Two new triterpenes from basidiomata of the medicinal and edible mushroom, *Laetiporus sulphureus*. Molecules.

[18] Hardt I.H., Jensen P.R., Fenical W. (2000). Neomarinone, and new cytotoxic marinone derivatives, produced by a marine filamentous bacterium (Actinomycetales). Tetrahedron Lett..

[19] Kalaitzis J.A., Hamano Y., Nilsen G., Moore B.S. (2003). Biosynthesis and structural revision of neomarinone. Org. Lett..

[20] Charan R.D., Schlingmann G., Bernan V.S., Feng X., Carter G.T. (2005). Fumaquinone, a new prenylated naphthoquinone from *Streptomyces fumanus*. J. Antibiot..

[21] Masuka A., Decock C., Mossebo D., Ryvarden L. (2022). Poroid Fungi of Africa.

[22] Yarza P., Yilmaz P., Pruesse E., Glöckner F.O., Ludwig W., Schleifer K.-H., Whitman W.B., Euzéby J., Amann R., Rosselló-Móra R. (2014). Uniting the classification of cultured and uncultured bacteria and archaea using 16S rRNA gene sequences. Nat. Rev. Microbiol..

[23] Song Y., Huang H., Chen Y., Ding J., Zhang Y., Sun A., Zhang W., Ju J. (2013). Cytotoxic and antibacterial marfuraquinocins from the deep south china sea-derived *Streptomyces niveus* scsio 3406. J. Nat. Prod..

[24] Jenner M., Jian X., Dashti Y., Masschelein J., Hobson C., Roberts D.M., Jones C., Harris S., Parkhill J., Raja H.A. (2019). An unusual: *Burkholderia gladioli* double chain-initiating nonribosomal peptide synthetase assembles ″fungal″ icosalide antibiotics. Chem. Sci..

[25] Fujishima M., Kodama Y. (2012). Endosymbionts in *Paramecium*. Eur. J. Protistol..

[26] Schüβler A., Schwarzott D., Walker C. (2001). A new fungal phylum, the Glomeromycota: Phylogeny and evolution. Mycol. Res..

[27] Cruz A.F., Horii S., Ochiai S., Yasuda A., Ishii T. (2008). Isolation and analysis of bacteria associated with spores of *Gigaspora margarita*. J. Appl. Microbiol..

[28] Bianciotto V., Lumini E., Bonfante P., Vandamme P. (2003). “*Candidatus Glomeribacter gigasporarum*” gen. nov., sp. nov., an endosymbiont of arbuscular mycorrhizal fungi. Int. J. Syst. Evol. Microbiol..

[29] Ryan M.P., Adley C.C. (2014). *Ralstonia* spp.: Emerging global opportunistic pathogens. Eur. J. Clin. Microbiol. Infect. Dis..

[30] Guo Y., Narisawa K. (2018). Fungus-bacterium symbionts promote plant health and performance. Microbes Environ..

[31] Zhang Y., Qiu S. (2016). Phylogenomic analysis of the genus *Ralstonia* based on 686 single-copy genes. Antonie Leeuwenhoek.

[32] Ryan M.P., Pembroke J.T., Adley C.C. (2007). *Ralstonia pickettii* in environmental biotechnology: Potential and applications. J. Appl. Microbiol..

[33] Ferro P., Vaz-Moreira I., Manaia C.M. (2019). Association between gentamicin resistance and stress tolerance in water isolates of *Ralstonia pickettii* and *R. mannitolilytica*. Folia Microbiol..

[34] Hassan E.A., Balabel N.M., Ahmed A.E., Eid N.A., Ramadan E.M. (2017). Relationship between *Ralstonia solanacearum* and bioagents recovered from different habitats. Int. J. Sci. Eng. Res..

[35] Bonfante P. (2003). Plants, mycorrhizal fungi and endobacteria: A dialog among cells and genomes. Biol. Bull..

[36] Bertaux J., Schmid M., Prevost-Boure N.C., Churin J.L., Hartmann A., Garbaye J., Frey-Klett P. (2003). In situ identification of intracellular bacteria related to *Paenibacillus* spp. in the mycelium of the ectomycorrhizal fungus *Laccaria bicolor* S238N. Appl. Environ. Microbiol..

[37] Aslani M.A., Harighi B., Abdollahzadeh J. (2018). Screening of endofungal bacteria isolated from wild growing mushrooms as potential biological control agents against brown blotch and internal stipe necrosis diseases of *Agaricus bisporus*. Biol. Control.

[38] Sharma M., Schmid M., Rothballer M., Hause G., Zuccaro A., Imani J., Kämpfer P., Domann E., Schäfer P., Hartmann A. (2008). Detection and identification of bacteria intimately associated with fungi of the order *Sebacinales*. Cell. Microbiol..

[39] Partida-Martinez L.P., Hertweck C. (2005). Pathogenic fungus harbours endosymbiotic bacteria for toxin production. Nature.

[40] Partida-Martinez L.P., De Looß C.F., Ishida K., Ishida M., Roth M., Buder K., Hertweck C. (2007). Rhizonin, the first mycotoxin isolated from the Zygomycota, is not a fungal metabolite but is produced by bacterial endosymbionts. Appl. Environ. Microbiol..

[41] Mosse B. (1970). Honey-coloured, sessile endogone spores: II. Changes in fine structure during spore development. Arch. Mikrobiol..

[42] Guo H., Glaeser S.P., Alabid I., Imani J., Haghighi H., Kämpfer P., Kogel K.-H. (2017). The abundance of endofungal bacterium *Rhizobium radiobacter* (syn. *Agrobacterium tumefaciens*) increases in its fungal host *Piriformospora indica* during the tripartite sebacinalean symbiosis with higher plants. Front. Microbiol..

[43] Kobayashi D.Y., Crouch J.A. (2009). Bacterial/fungal interactions: From pathogens to mutualistic endosymbionts. Annu. Rev. Phytopathol..

[44] Alabid I., Glaeser S.P., Kogel K.-H. (2019). Endofungal bacteria increase fitness of their host fungi and impact their association with crop plants. Curr. Issues Mol. Biol..

[45] Pent M., Bahram M., Põldmaa K. (2020). Fruitbody chemistry underlies the structure of endofungal bacterial communities across fungal guilds and phylogenetic groups. ISME J..

[46] Lackner G., Hertweck C. (2011). Impact of endofungal bacteria on infection biology, food safety, and drug development. PLoS Pathog..

[47] Lackner G., Partida-Martinez L.P., Hertweck C. (2009). Endofungal bacteria as producers of mycotoxins. Trends Microbiol..

[48] Spraker J.E., Sanchez L.M., Lowe T.M., Dorrestein P.C., Keller N.P. (2016). *Ralstonia solanacearum* lipopeptide induces chlamydospore development in fungi and facilitates bacterial entry into fungal tissues. ISME J..

