# Supplementary material for: Meroterpenoids Possibly Produced by a Bacterial Endosymbiont of the Tropical Basidiomycete Echinochaete brachypora
Source: Biomolecules. 2022 May 28;12(6):755. doi: 10.3390/biom12060755 (PMC9221130; doi:10.3390/biom12060755)
Supplement: Supplementary file 1 [file biomolecules-12-00755-s001.zip › biomolecules-1719305-supplementary.pdf]

Meroterpenoids possibly produced by a bacterial endosymbiont of the tropical basidiomycete *Echinochaete brachypora*

Khadija Hassan[1,2], Clara Chepkirui[1,3], Natalia Andrea Llanos-López[1,2], Josphat C. Matasyoh [4], Cony Decock[5], Yasmina Marin-Felix[1,2,*] and Marc Stadler[1,2*]

[1] Department Microbial Drugs, Helmholtz Centre for Infection Research (HZI), German Centre for InfectionResearch (DZIF), Partner Site Hannover/Braunschweig, Inhoffenstrasse 7, 38124 Braunschweig, Gemany
[2] Institute of Microbiology, Technische Universität Braunschweig, Spielmannstraße 7, 38106 Braunschweig, Germany khadija.hassan@helmholtz-hzi.de ; yasmina.marinfelix@helmholtz-hzi.de ; natalia.llanos@helmholtz-hzi.de
[3] Swiss Federal Institute of Technology in Zurich (ETH) cchepkirui@biol.ethz.ch ;
[3] Egerton University, Department of Chemistry, P.O BOX 536, 20115, Njoro, Kenya; josphat2001@yahoo.com
[4] Mycothéque de l' Universite catholique de Louvain (BCCM/MUCL), Place Croix du Sud 3, B-1348 Louvain-la-Neuve, Belgium ; cony.decock@uclouvain.be

Supplementary Information

Figure S1: ESIMS data for compound **1** 3
Figure S2: ^{1}H NMR spectrum (acetone-d$_4$, 500 MHz) of (**1**). 4
Figure S3: ^{13}C NMR spectrum (acetone-d_4, 700 MHz) of (1). 5
Figure S4: HSQC spectrum in acetone-d_4 (^{1}H 500 MHz, ^{13}C 125) of (**1**). 6
Figure S5: HMBC spectrum in acetone-d_6 (^{1}H 500 MHz, ^{13}C 125 MHz) of (**1**). 7
Figure S6: COSY spectrum in acetone-d_6 (^{1}H 500 MHz) of (**1**). 8
Figure S7: ROESY spectrum in acetone-d_6 (^{1}H 500 MHz) of (**1**). 9
Figure S8: ESIMS data for compound **2** 10
Figure S9: ^{1}H NMR spectrum (acetone-d_4, 500 MHz) of (2). 11
Figure S10: ^{13}C NMR spectrum (acetone-d_4, 700 MHz) of (2) 12
Figure S11: HSQC spectrum in acetone-d_4 (^{1}H 500 MHz, ^{13}C 125) of (**2**). 13
Figure S12: HMBC spectrum in acetone-d$_6$ (^{1}H 500 MHz, ^{13}C 125 MHz) of (**2**). 14

(https://creativecommons.org/licenses/by/4.0/).

Figure S13: ROESY spectrum in acetone-d_6 (^{1}H 500 MHz) of (**2**). 15
Figure S14: COSY spectrum in acetone-d_6 (^{1}H 500 MHz) of (**2**). 16
Figure S15: ESIMS data for compound **3** 17
Figure S16: ^{1}H NMR spectrum (acetone-d_4, 500 MHz) of (**3**). 18
Figure S17: ^{13}C NMR spectrum (acetone-d_4, 700 MHz) of (**3**). 19
Figure S18: HSQC spectrum in acetone-d_4 (^{1}H 500 MHz, ^{13}C 125) of (**3**). 20
Figure S19: HMBC spectrum in acetone-d_6 (^{1}H 500 MHz, ^{13}C 125 MHz) of (**3**). 21
Figure S20: COSY spectrum in acetone-d_6 (^{1}H 500 MHz) of (**3**). 22
Figure S21: ESIMS data for compound **4** 23
Figure S22: ^{1}H NMR spectrum (acetone-d_4, 500 MHz) of (**4**). 24
Figure S23: ^{13}C spectrum (acetone-d_4, ^{13}C 125) of **(4)** 25
Figure 24: HSQC spectrum in acetone-d_4 (^{1}H 500 MHz, ^{13}C 125) of **(4).** 26
Figure 25: HMBC spectrum in acetone-d_6 (^{1}H 500 MHz, ^{13}C 125 MHz) of (**4**). 27
Figure 26: ROESY spectrum in acetone-d_6 (^{1}H 500 MHz) of (**4**). 28
Figure S27: Phylogeny of Echinochaete, based on LSU and ITS 29

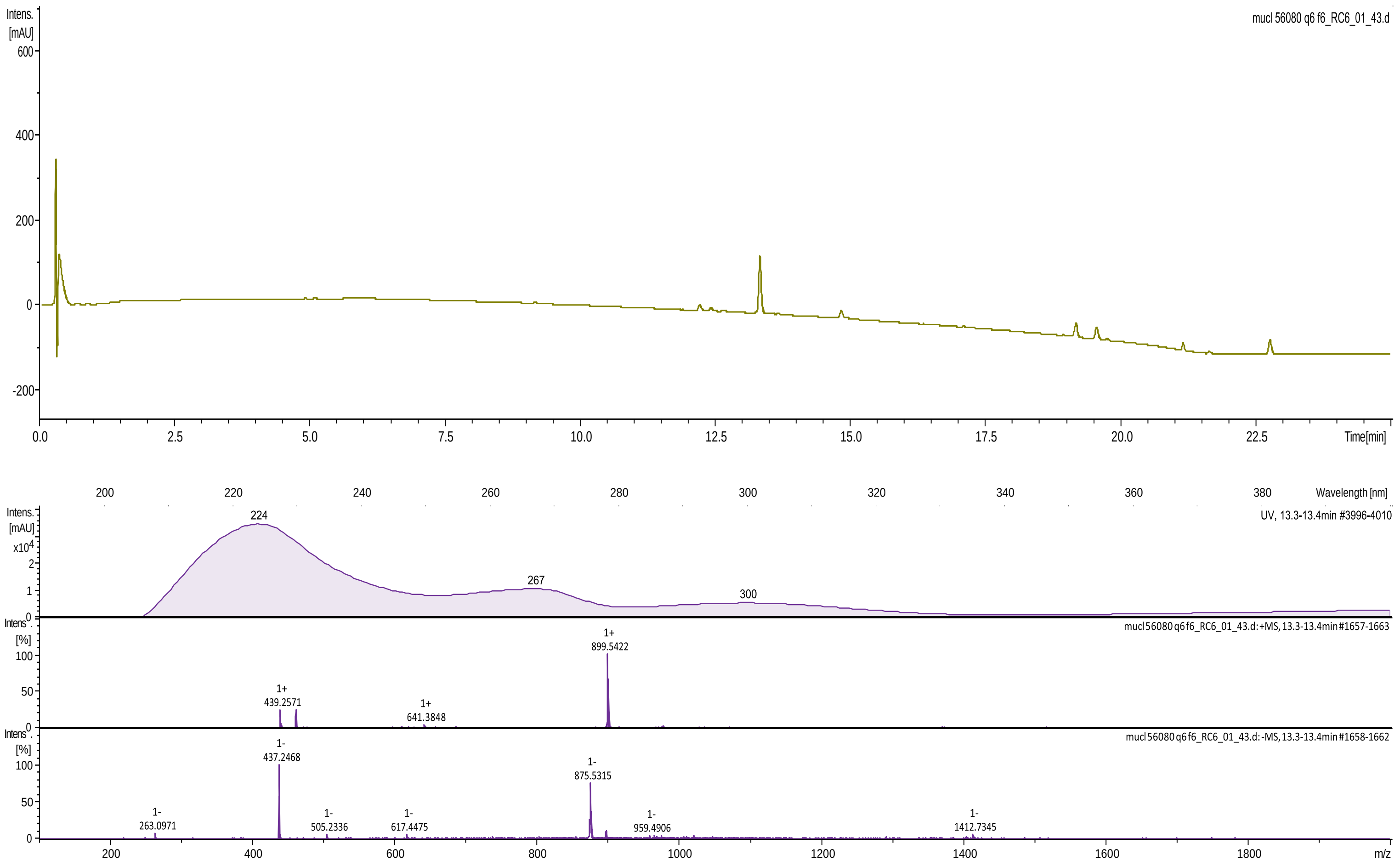

Figure S1: ESIMS data for compound **1**

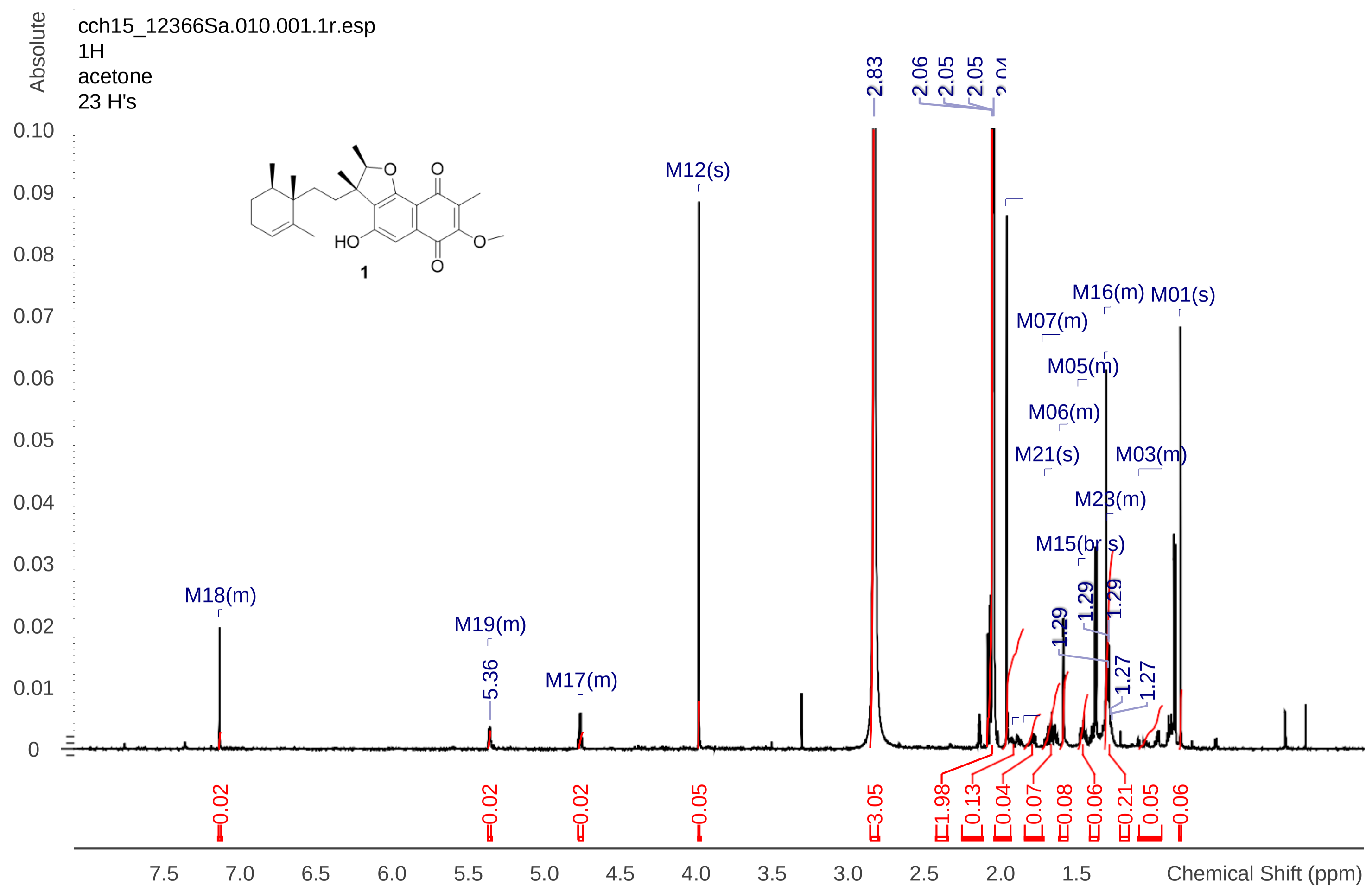

Figure S2: ^{1}H NMR spectrum (acetone-d_4, 500 MHz) of (**1**).

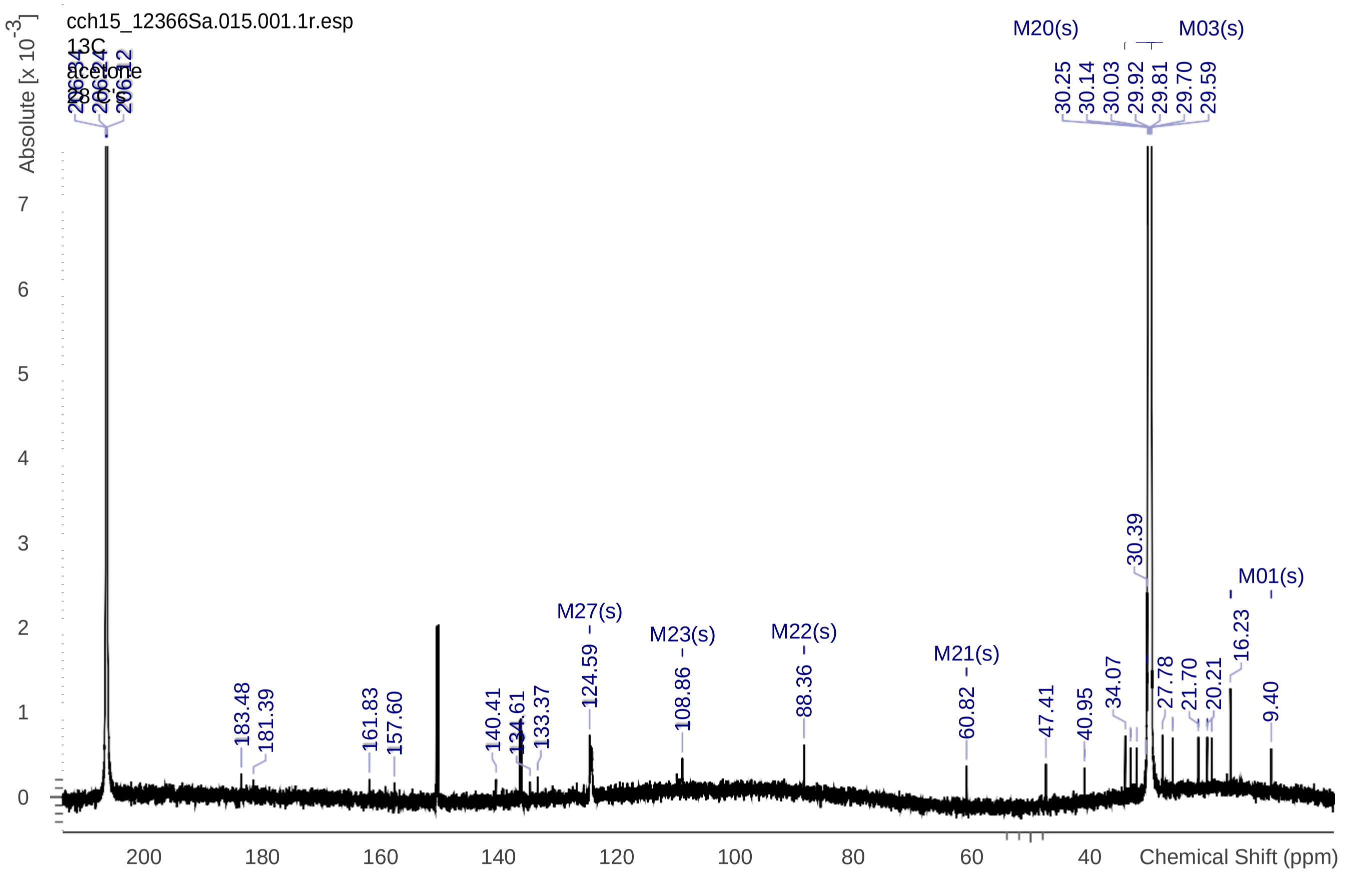

Figure S3: ^{13}C NMR spectrum (acetone-*d*$_4$, 700 MHz) of (1).

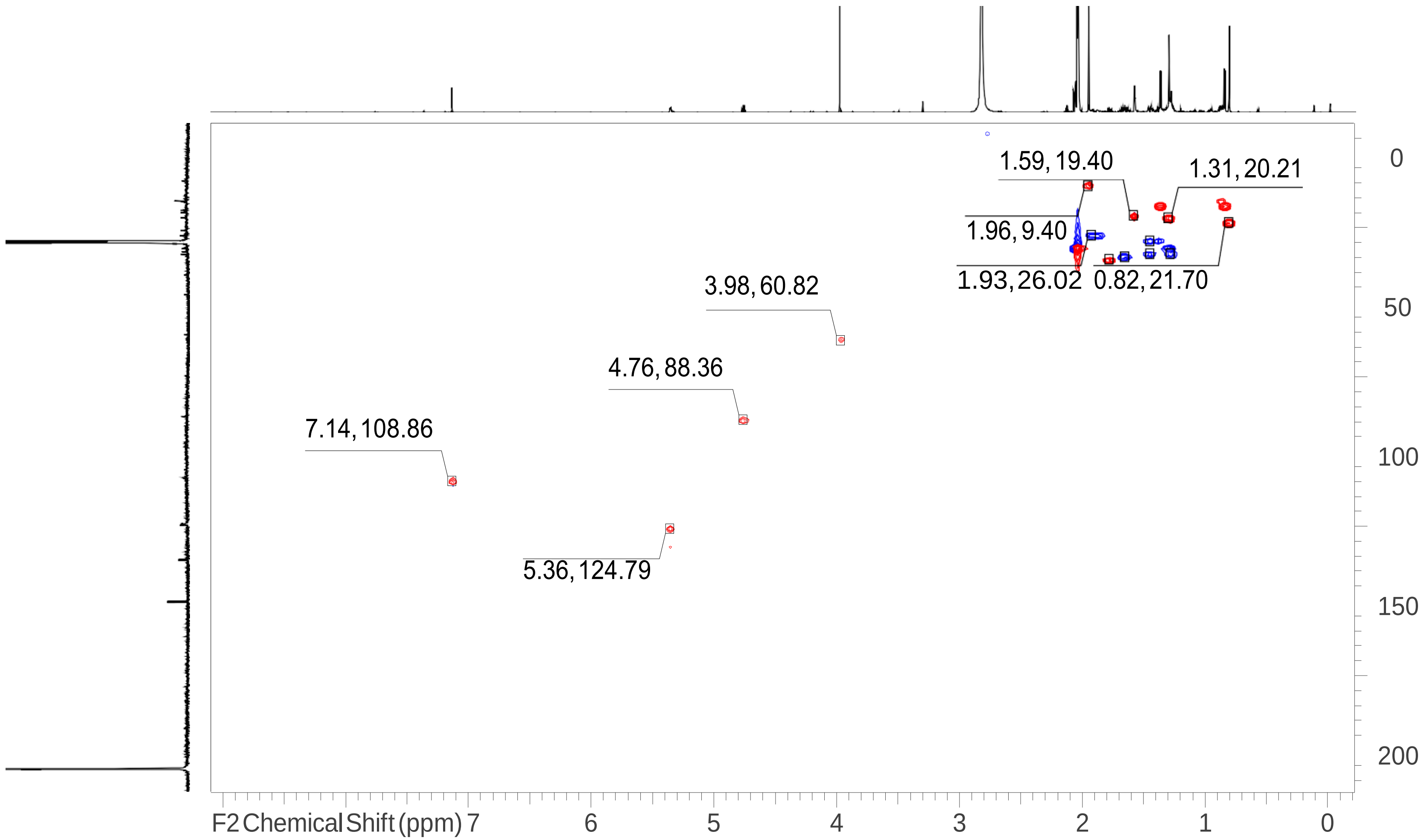

Figure S4: HSQC spectrum in acetone-d_4 (^{1}H 500 MHz, ^{13}C 125) of (**1**).

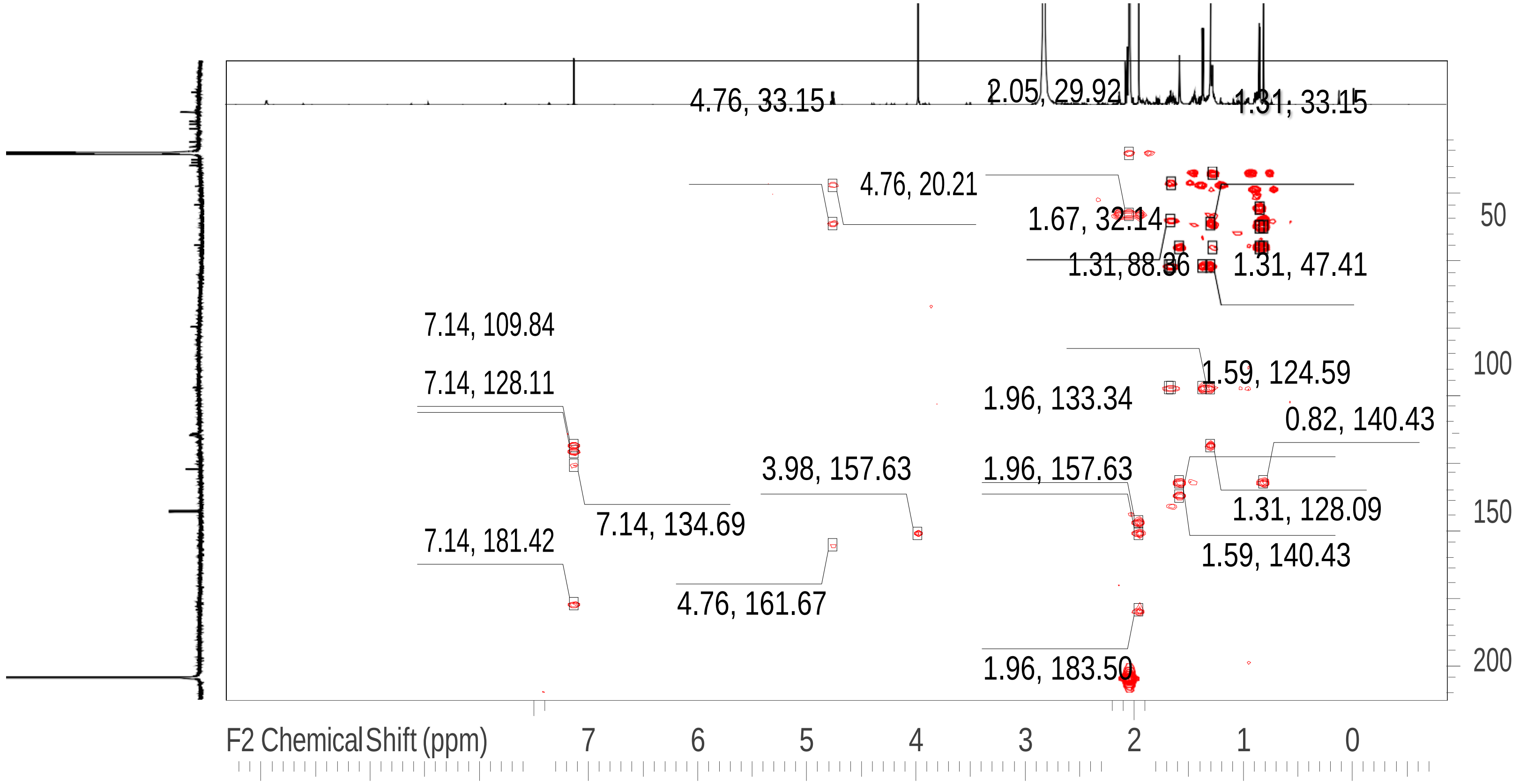

Figure S5: HMBC spectrum in acetone-d_6 (^{1}H 500 MHz, ^{13}C 125 MHz) of (**1**).

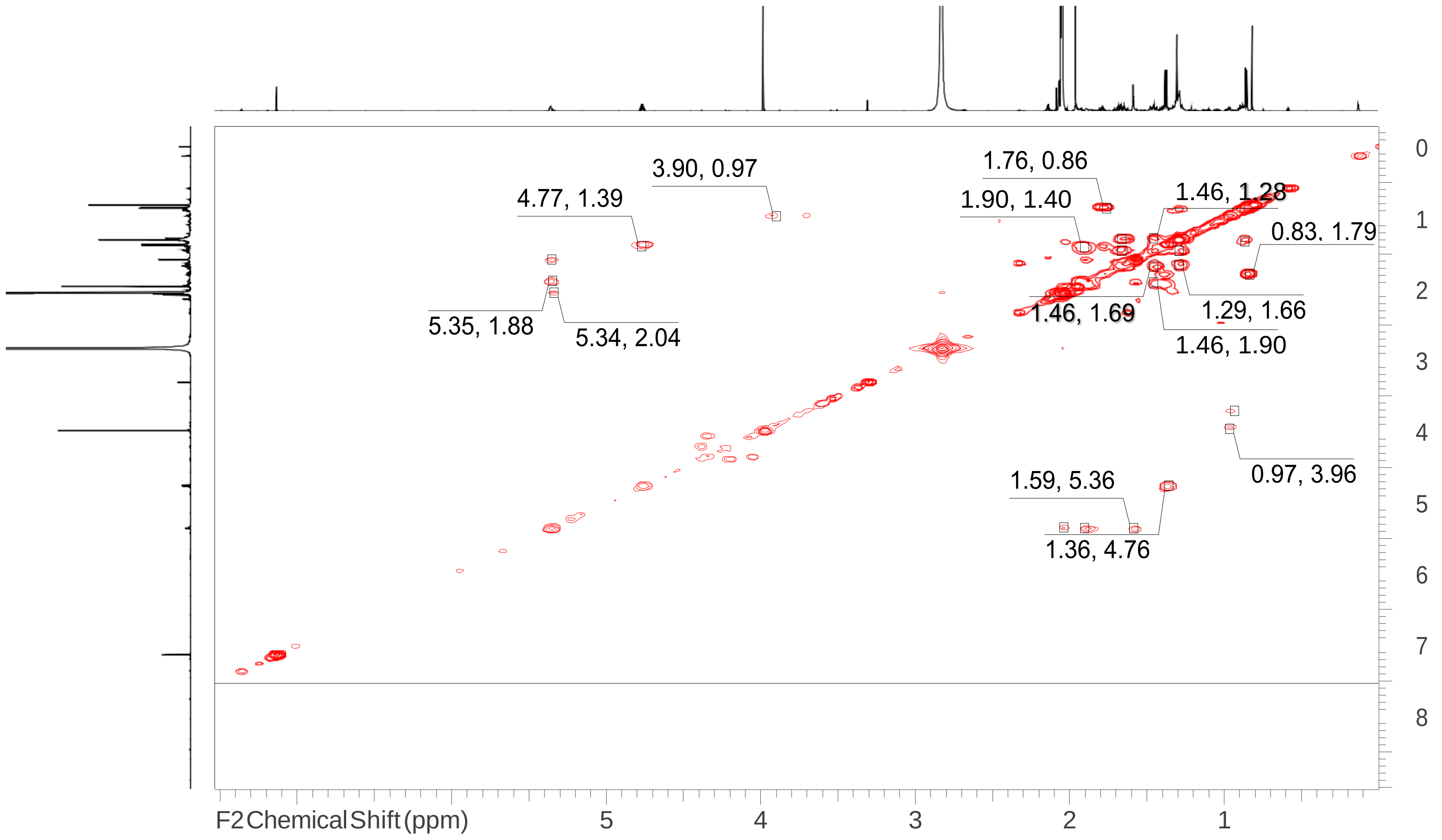

Figure S6: COSY spectrum in acetone-d_6 (^{1}H 500 MHz) of (**1**).

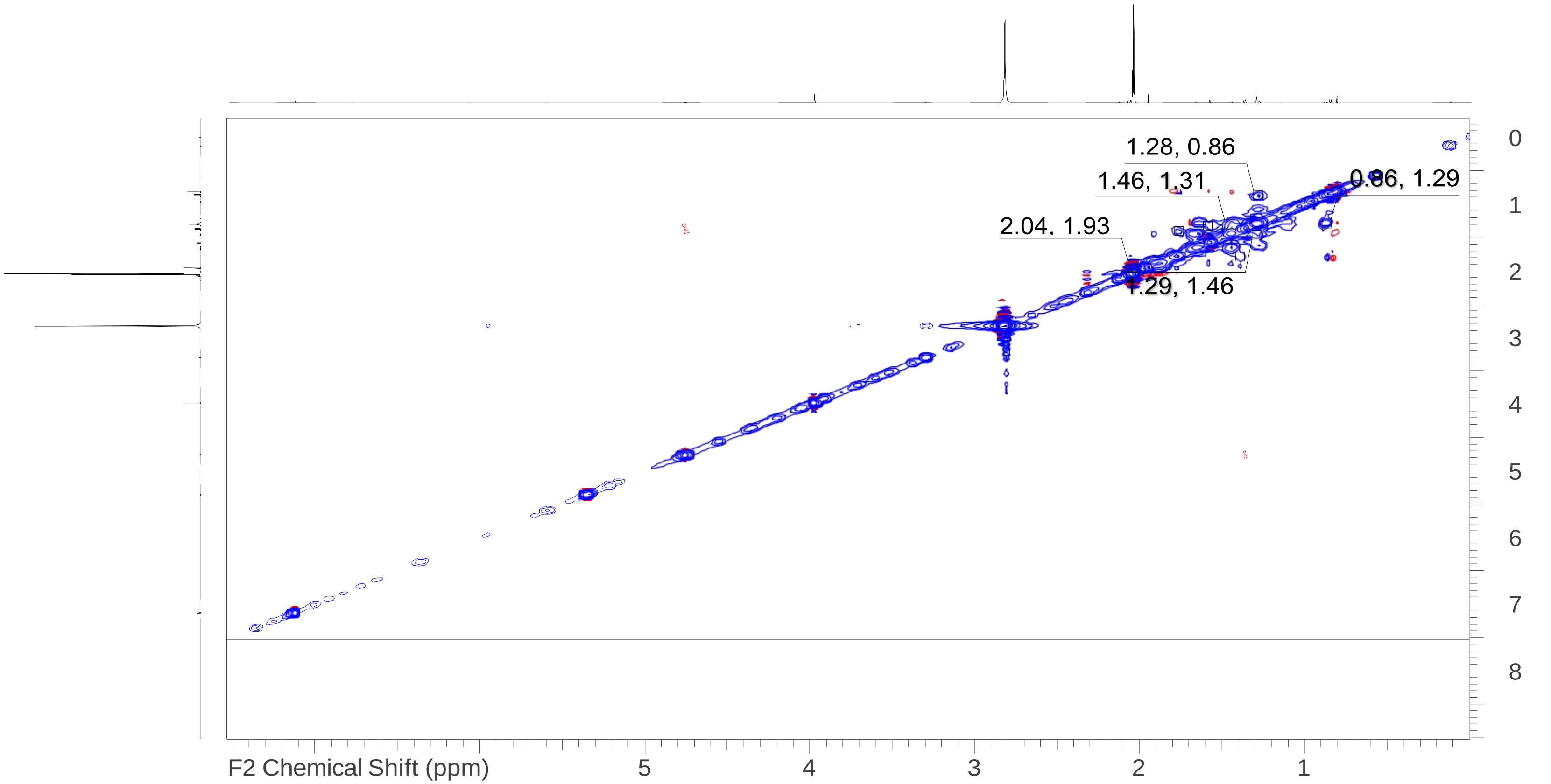

Figure S7: ROESY spectrum in acetone-d_6 (^{1}H 500 MHz) of (**1**).

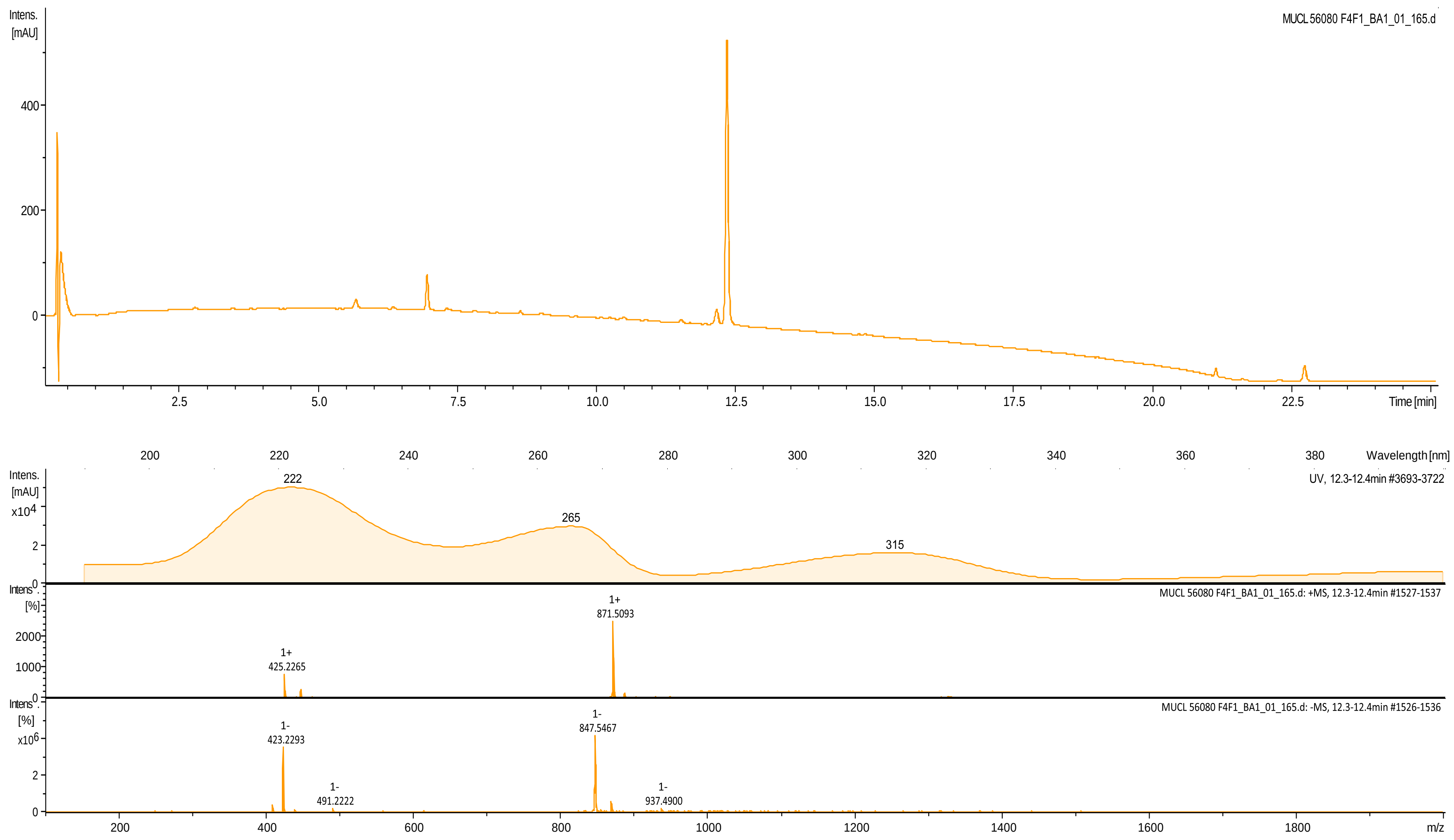

Figure S8: ESIMS data for compound **2**

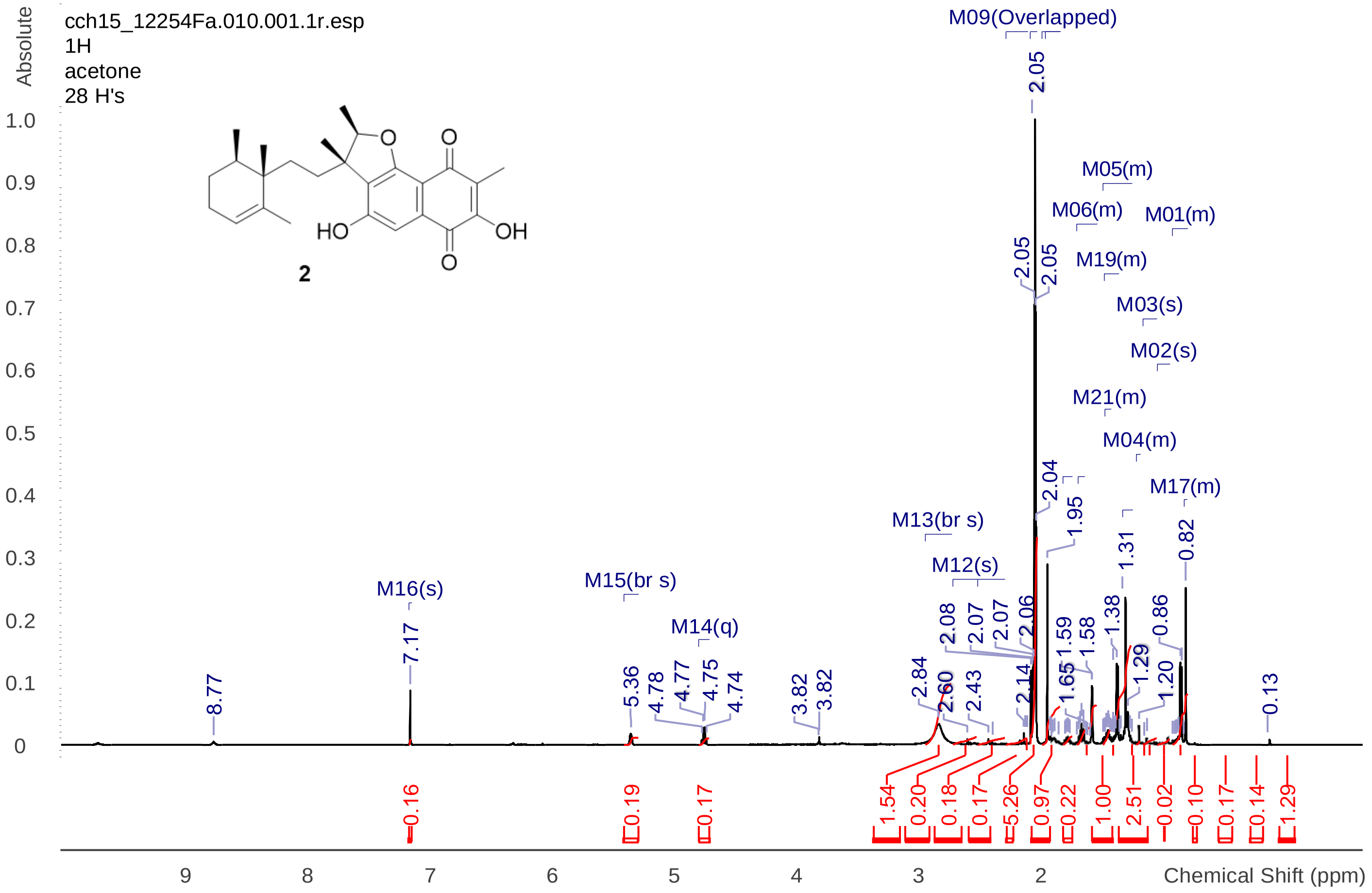

Figure S9: ^{1}H NMR spectrum (acetone-d_4, 500 MHz) of (2).

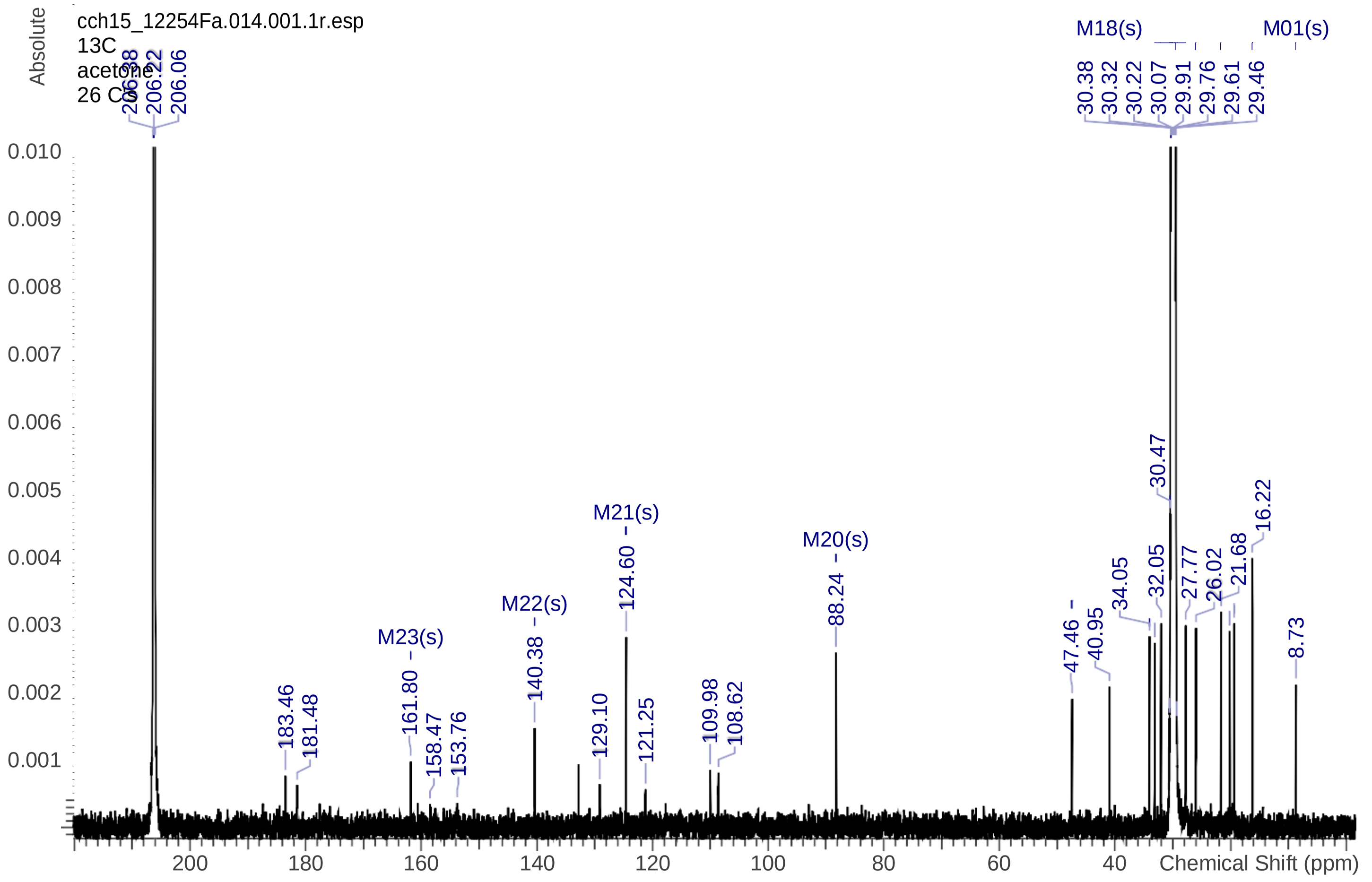

Figure S10: ^{13}C NMR spectrum (acetone-d_4, 700 MHz) of (2).

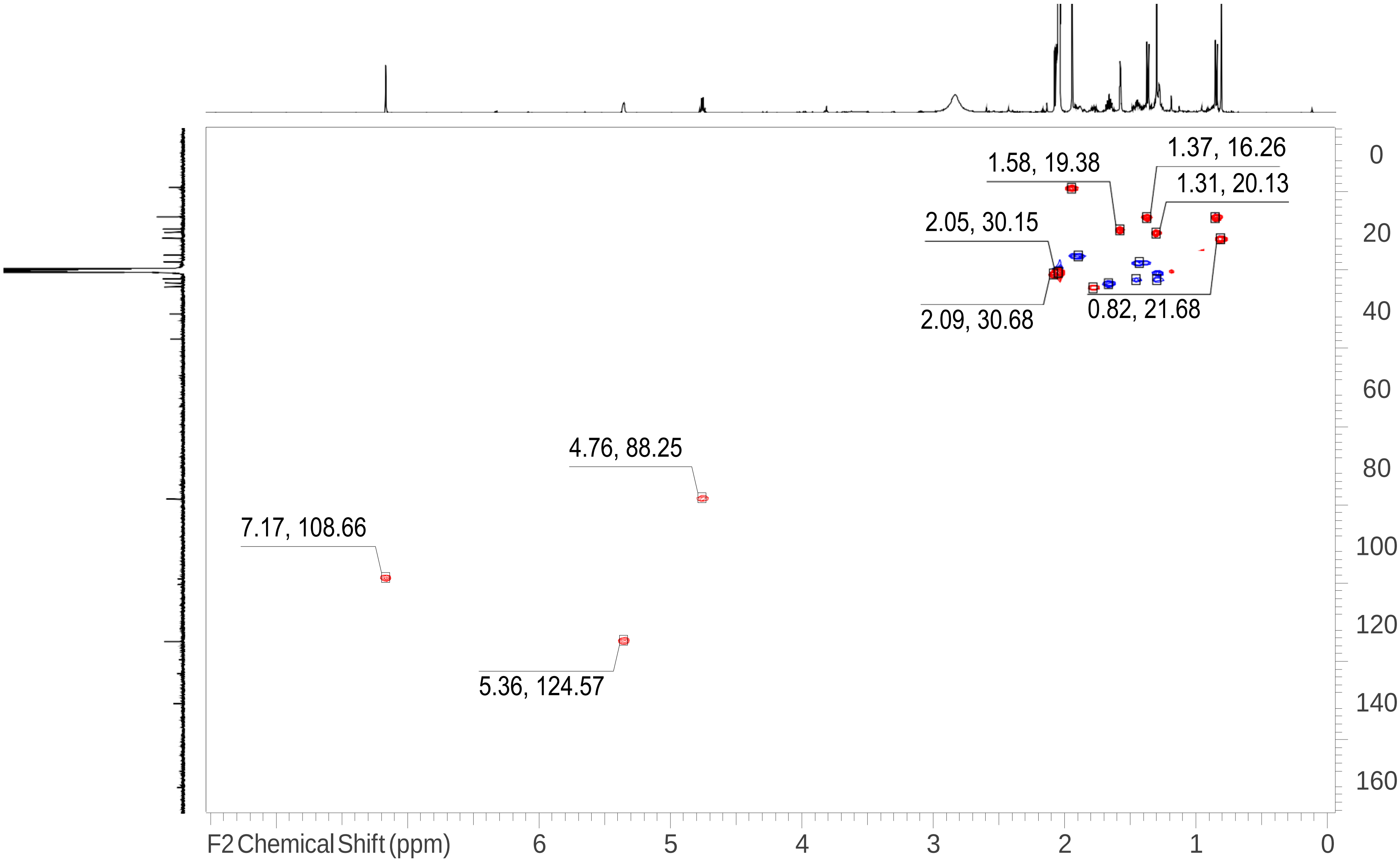

Figure S11: HSQC spectrum in acetone-*d*4 (^{1}H 500 MHz, ^{13}C 125) of (**2**).

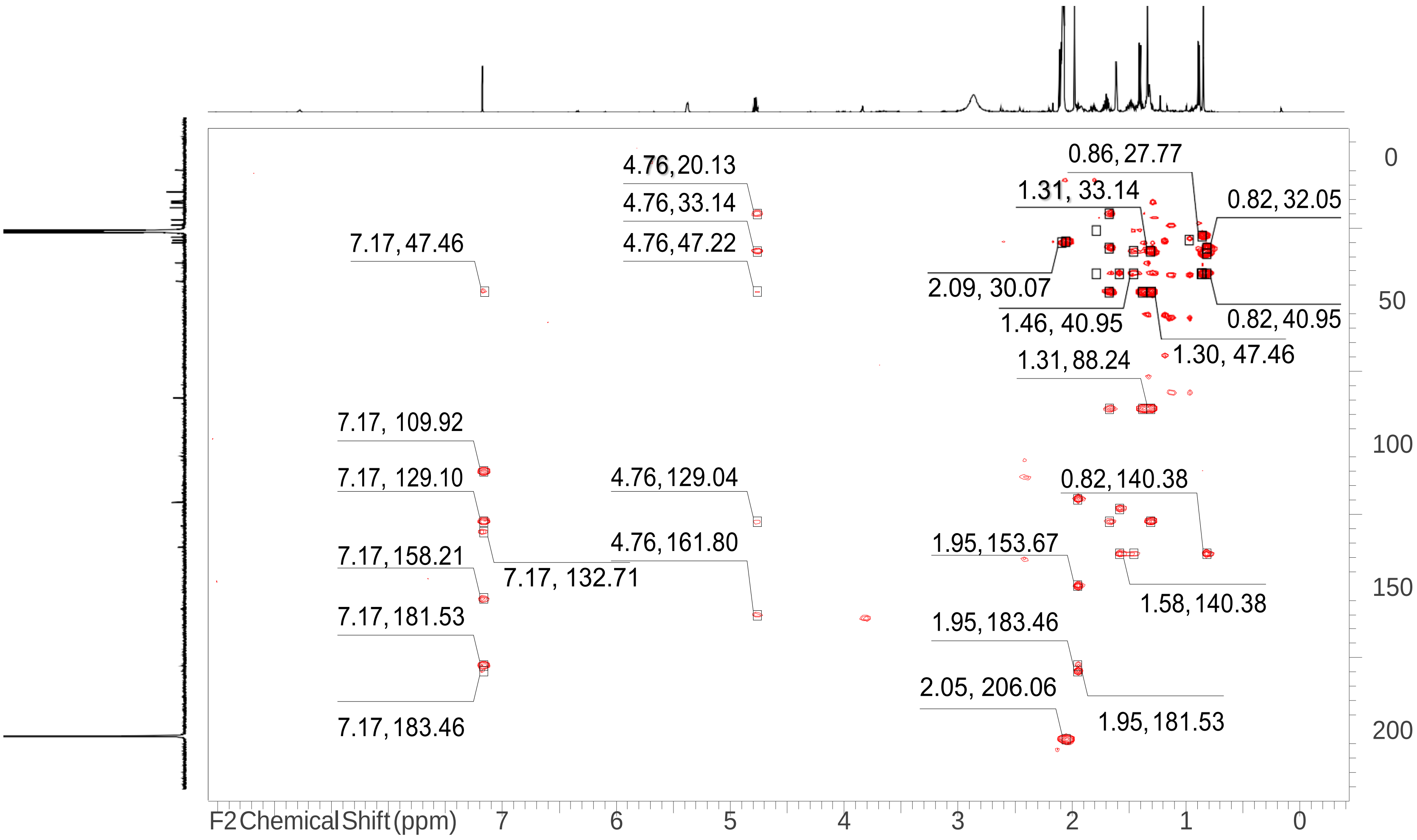

Figure S12: HMBC spectrum in acetone-d_6 (^{1}H 500 MHz, ^{13}C 125 MHz) of (**2**).

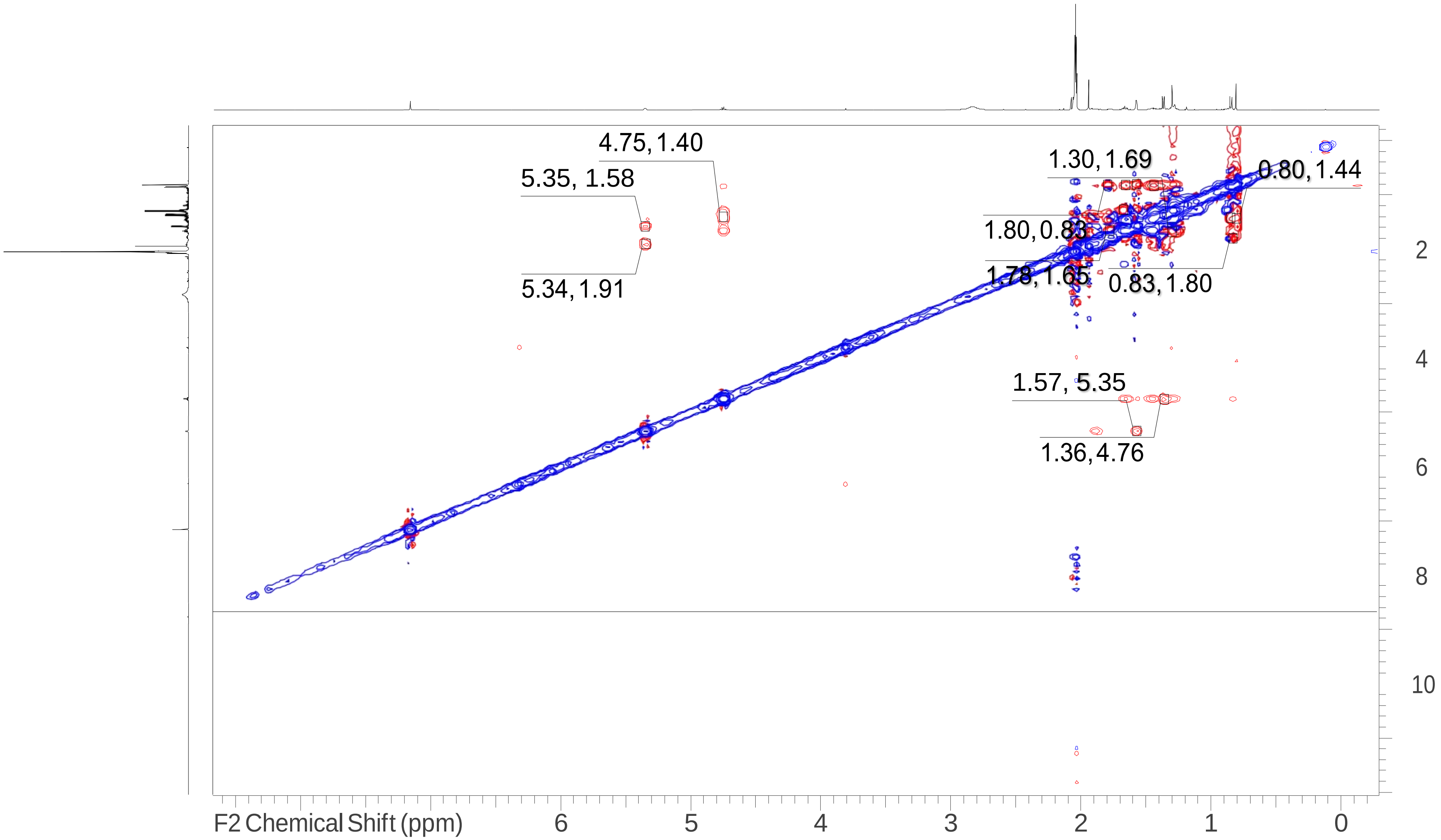

Figure S13: ROESY spectrum in acetone-d_6 (^{1}H 500 MHz) of (**2**).

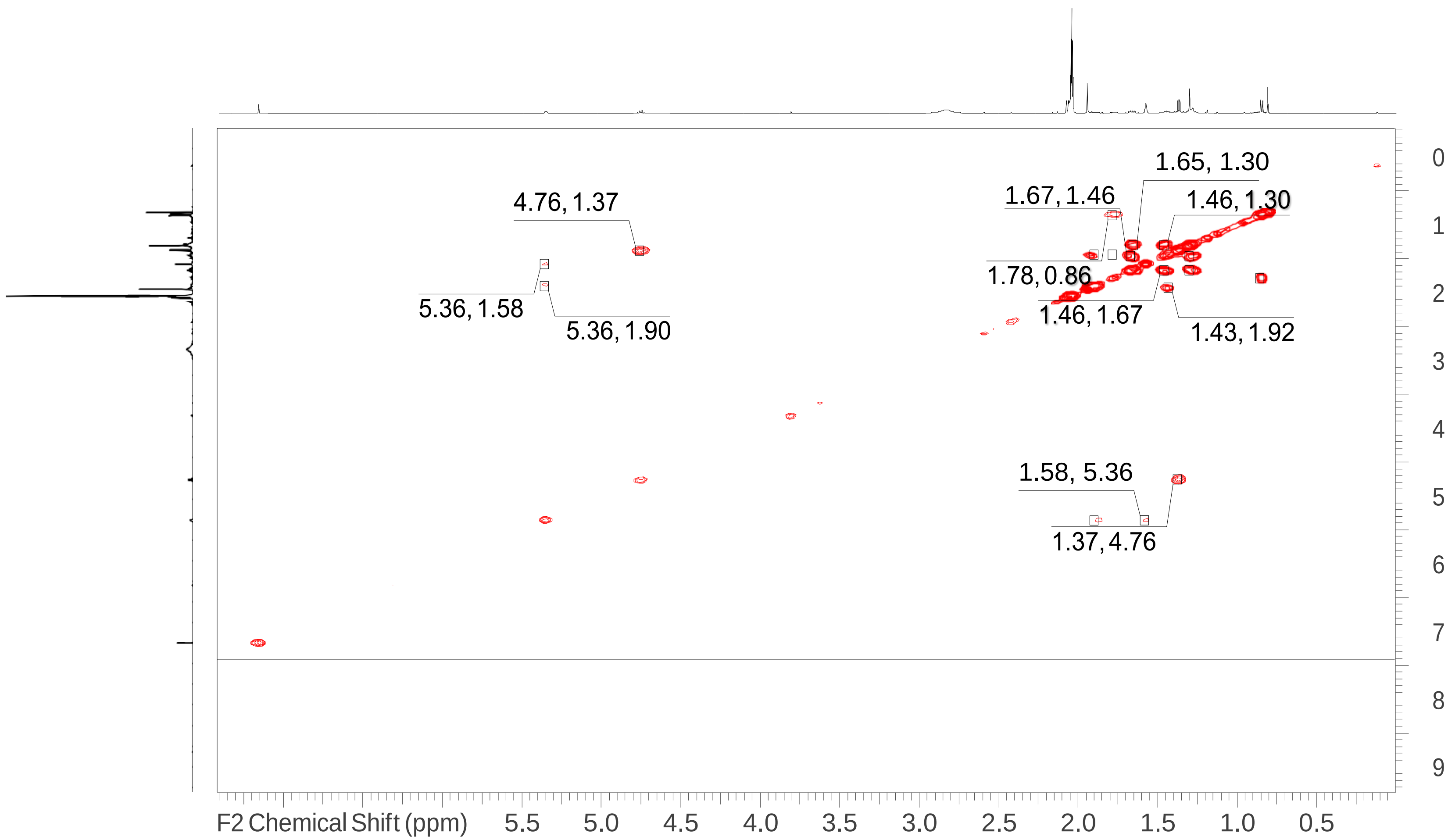

Figure S14: COSY spectrum in acetone-d_6 (^{1}H 500 MHz) of (**2**).

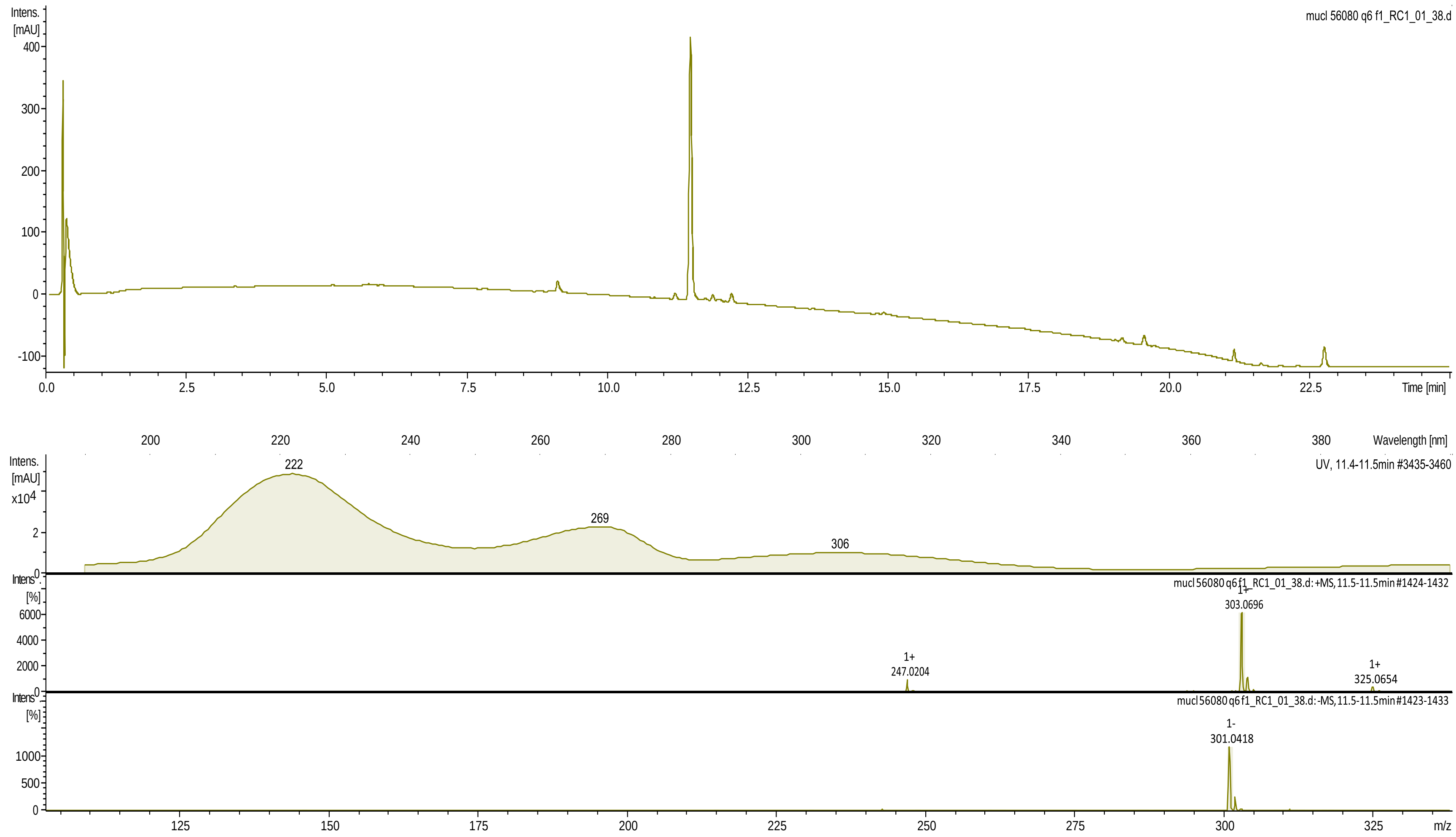

Figure S15: ESIMS data for compound **3**

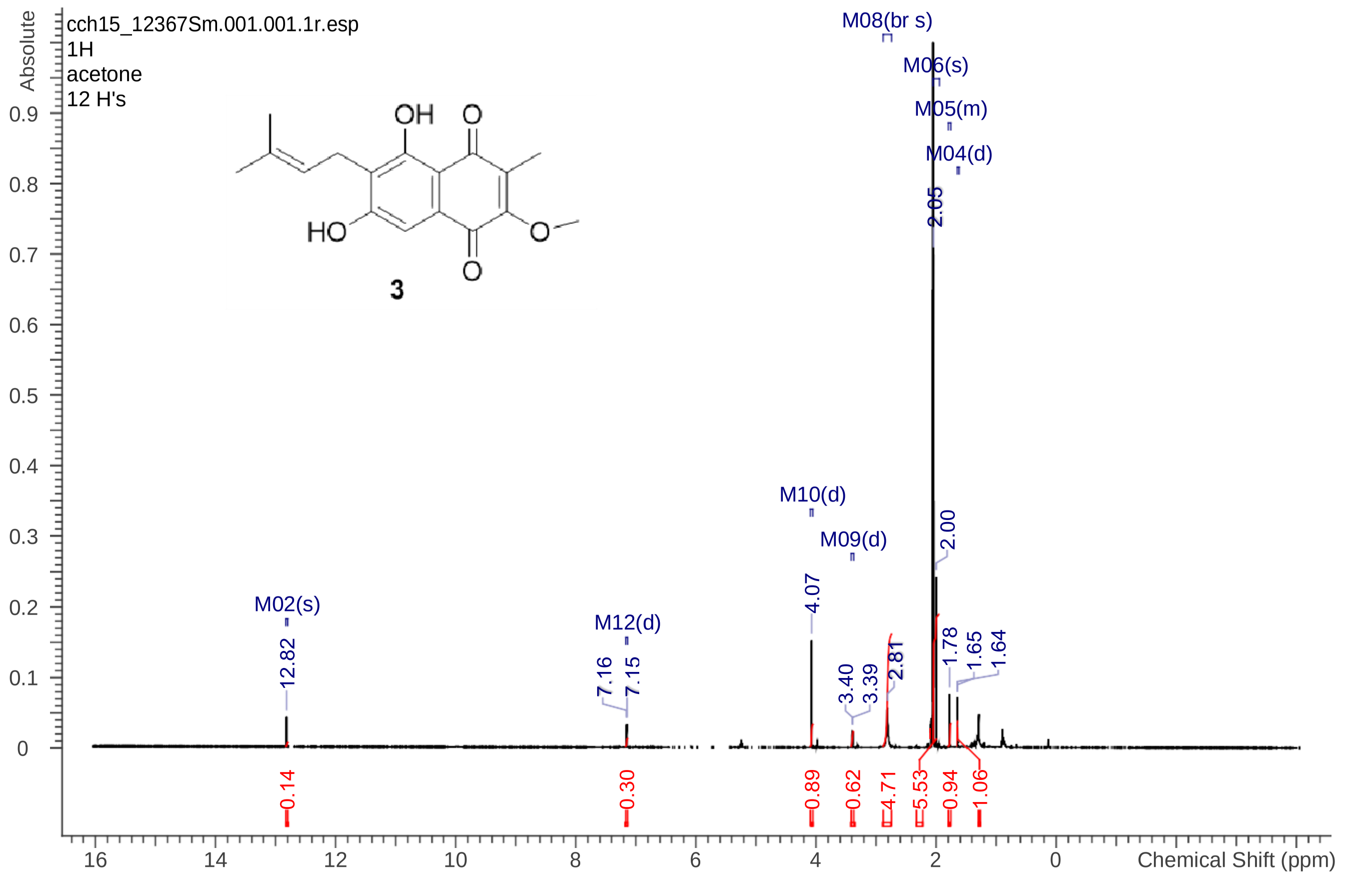

Figure S16: ^{1}H NMR spectrum (acetone-d_4, 500 MHz) of (**3**).

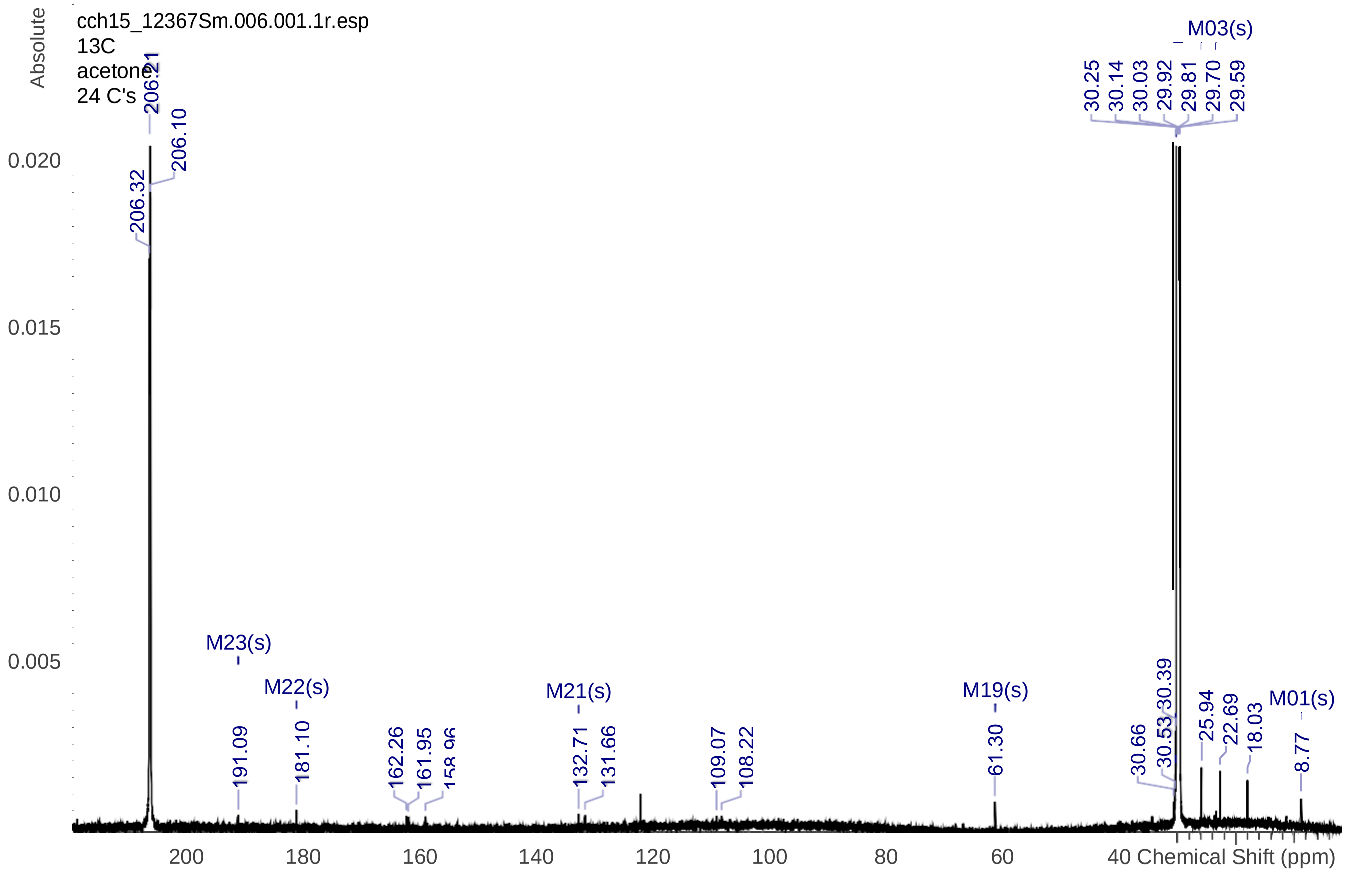

Figure S17: ^{13}C NMR spectrum (acetone-*d*$_4$, 700 MHz) of (**3**).

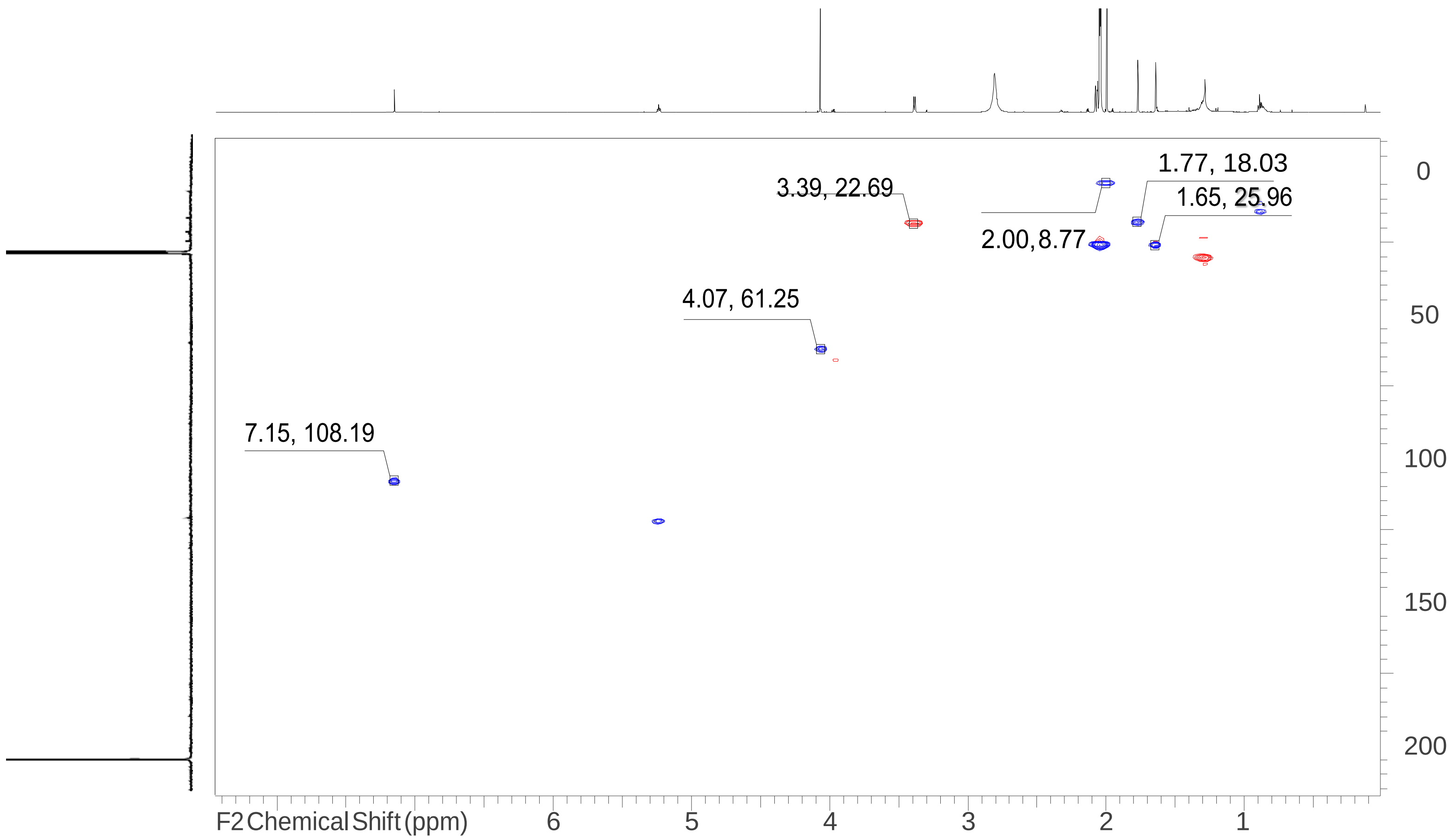

Figure S18: HSQC spectrum in acetone-d_4 (^{1}H 500 MHz, ^{13}C 125) of (**3**).

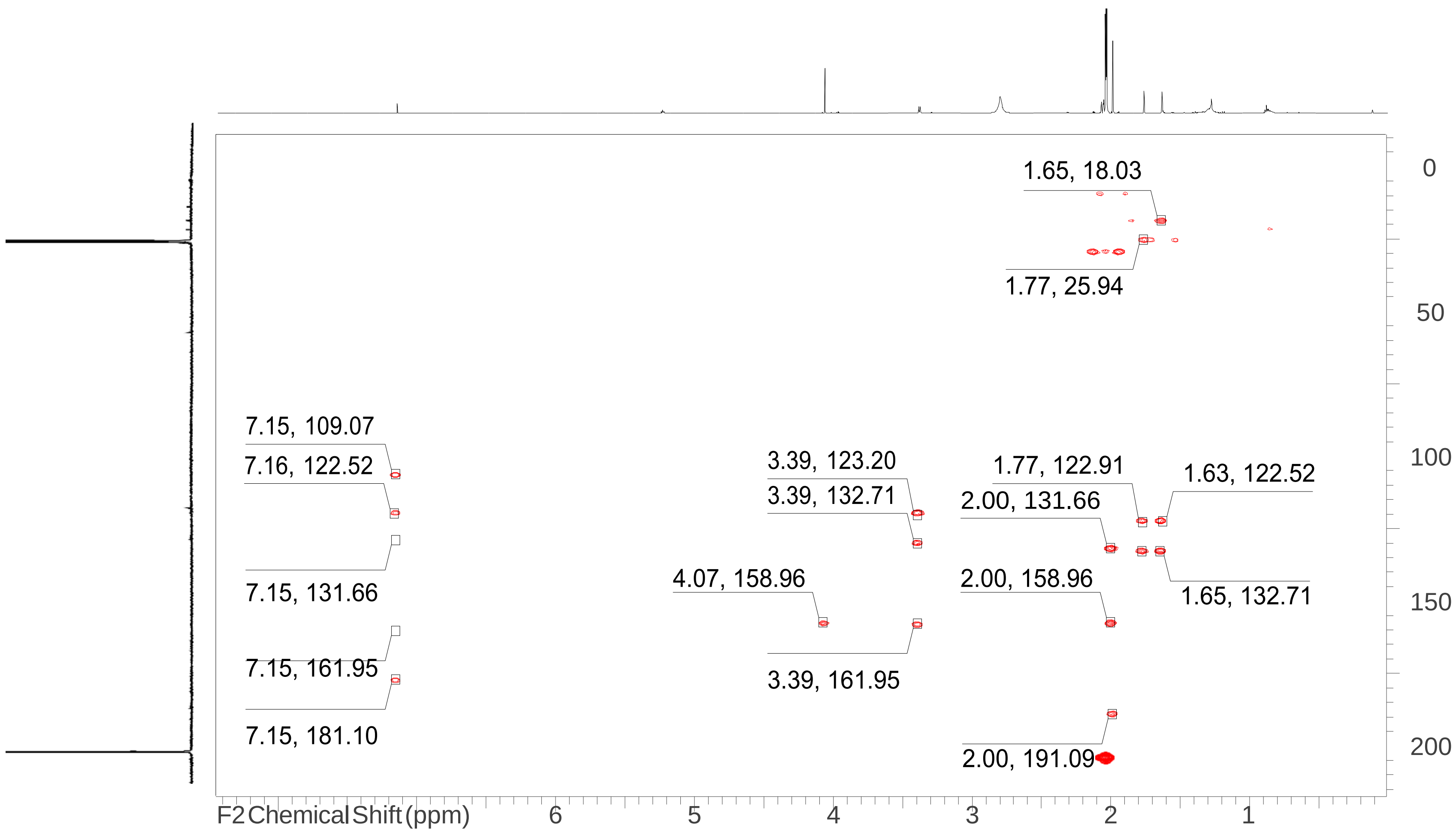

Figure S19: HMBC spectrum in acetone-d_6 (^{1}H 500 MHz, ^{13}C 125 MHz) of (**3**).

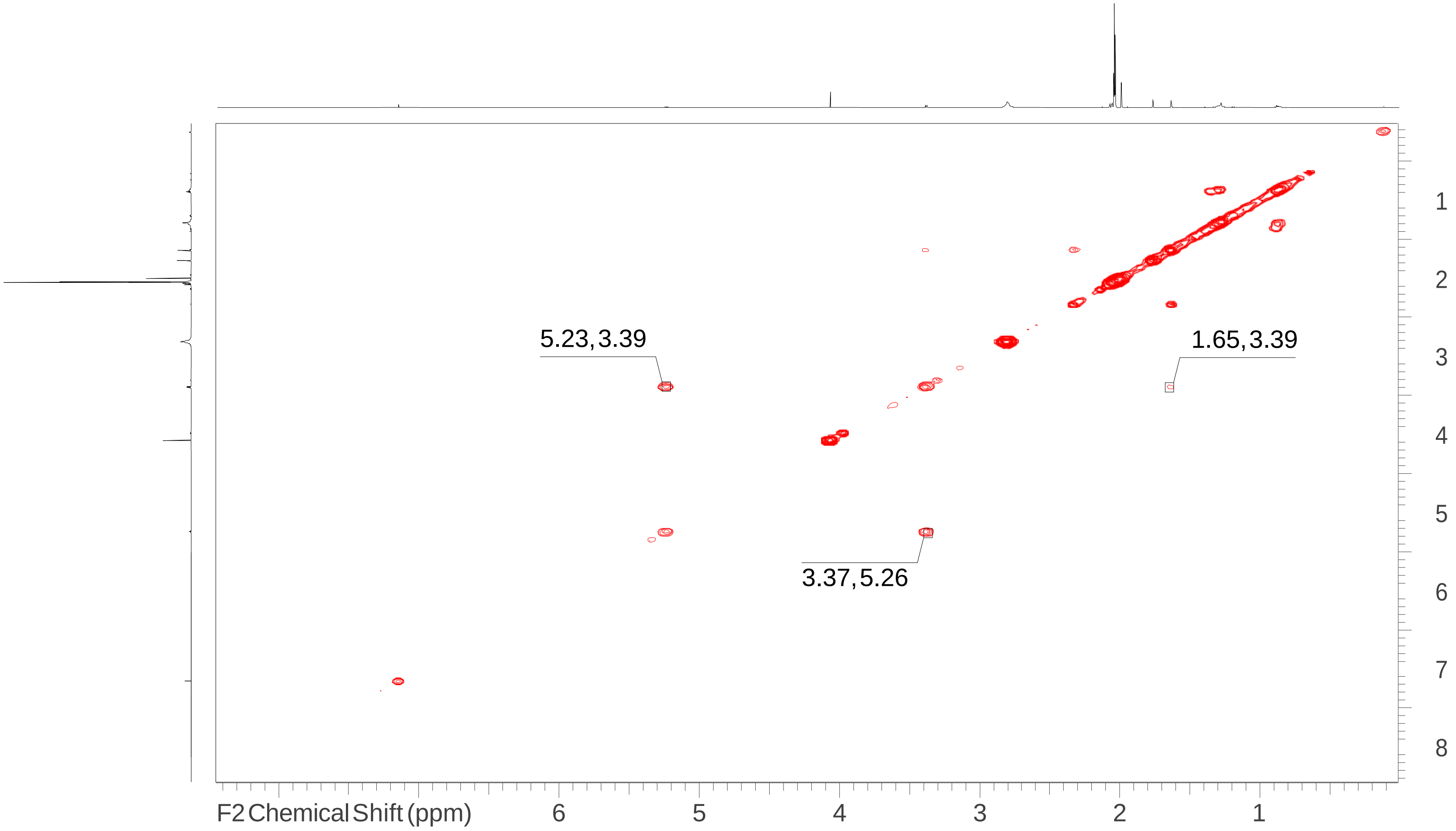

Figure S20: COSY spectrum in acetone-d_6 (^{1}H 500 MHz) of (**3**).

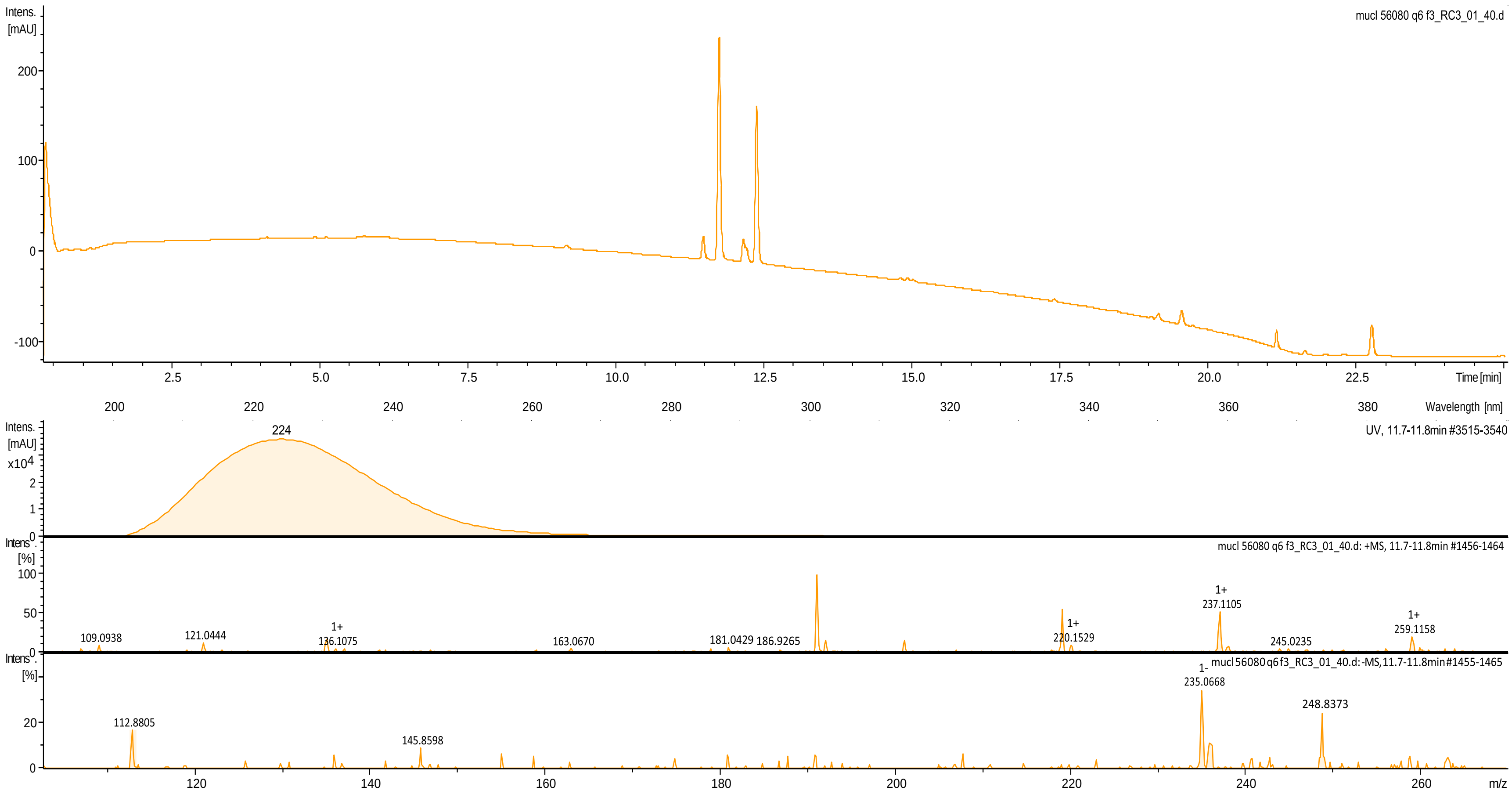

Figure S21: ESIMS data for compound **4**

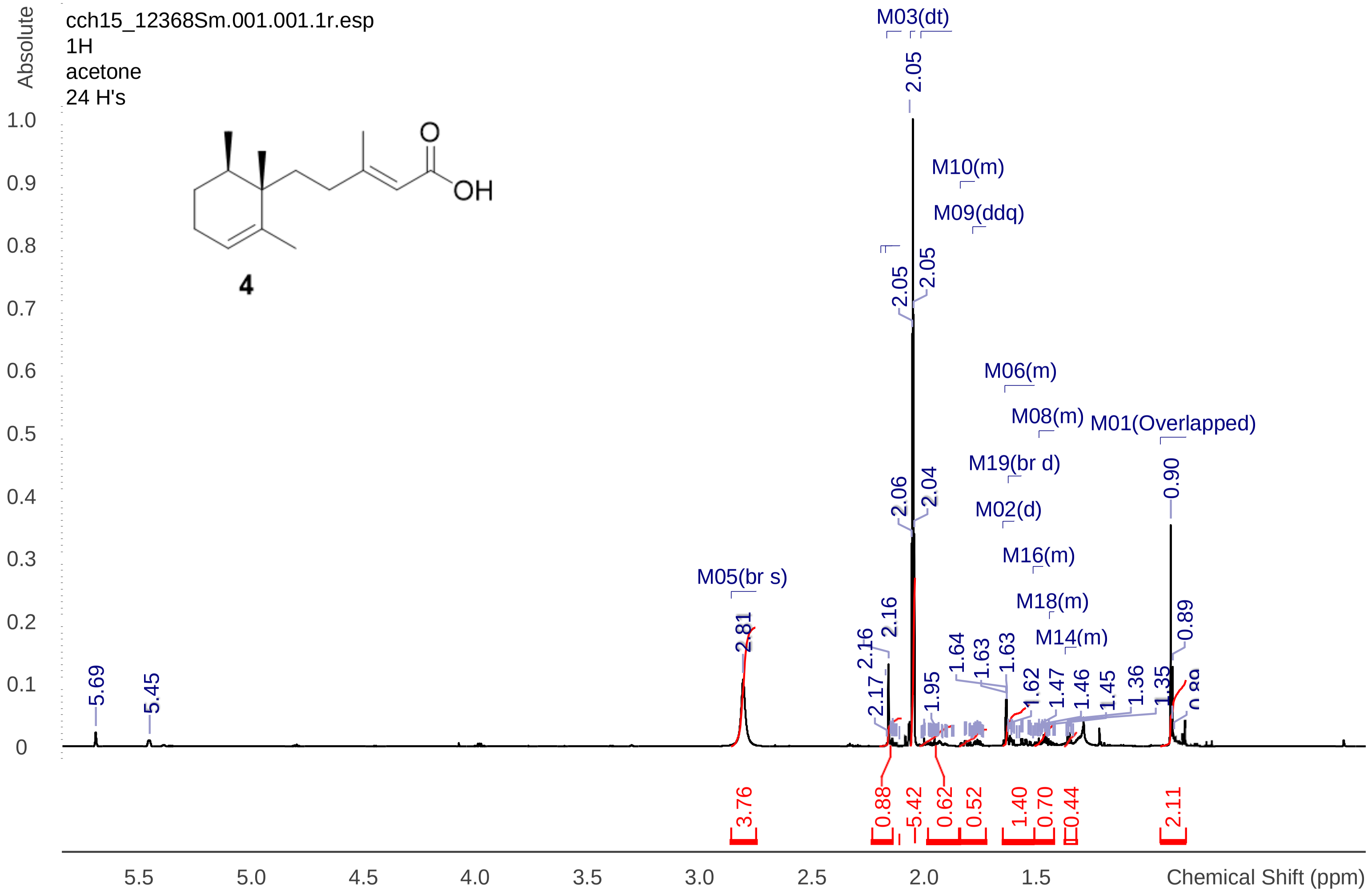

Figure S22: ^{1}H NMR spectrum (acetone-d_4, 500 MHz) of (**4**).

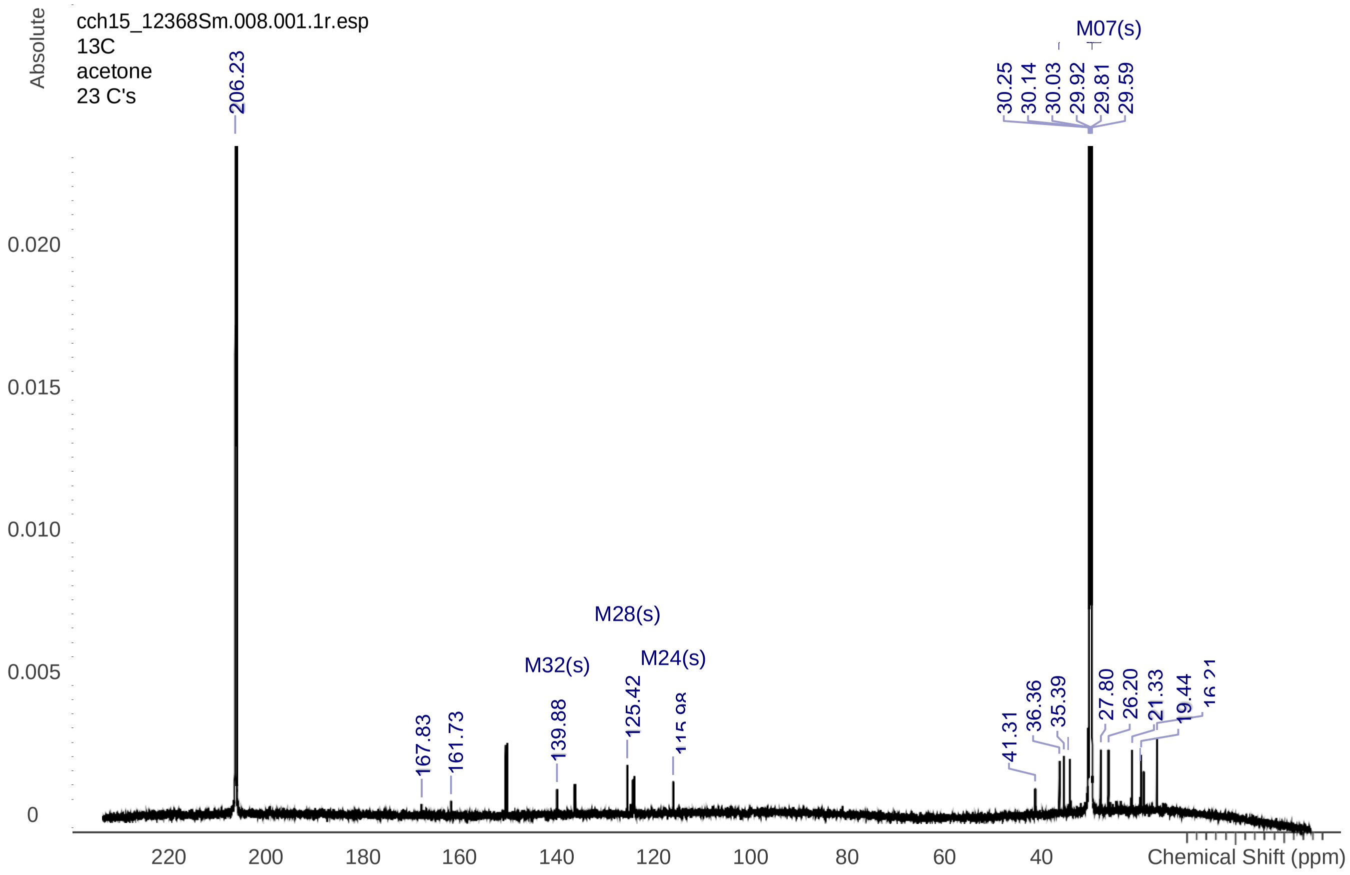

Figure S23: ^{13}C spectrum (acetone-*d*$_4$, ^{13}C 125) of **(4)**.

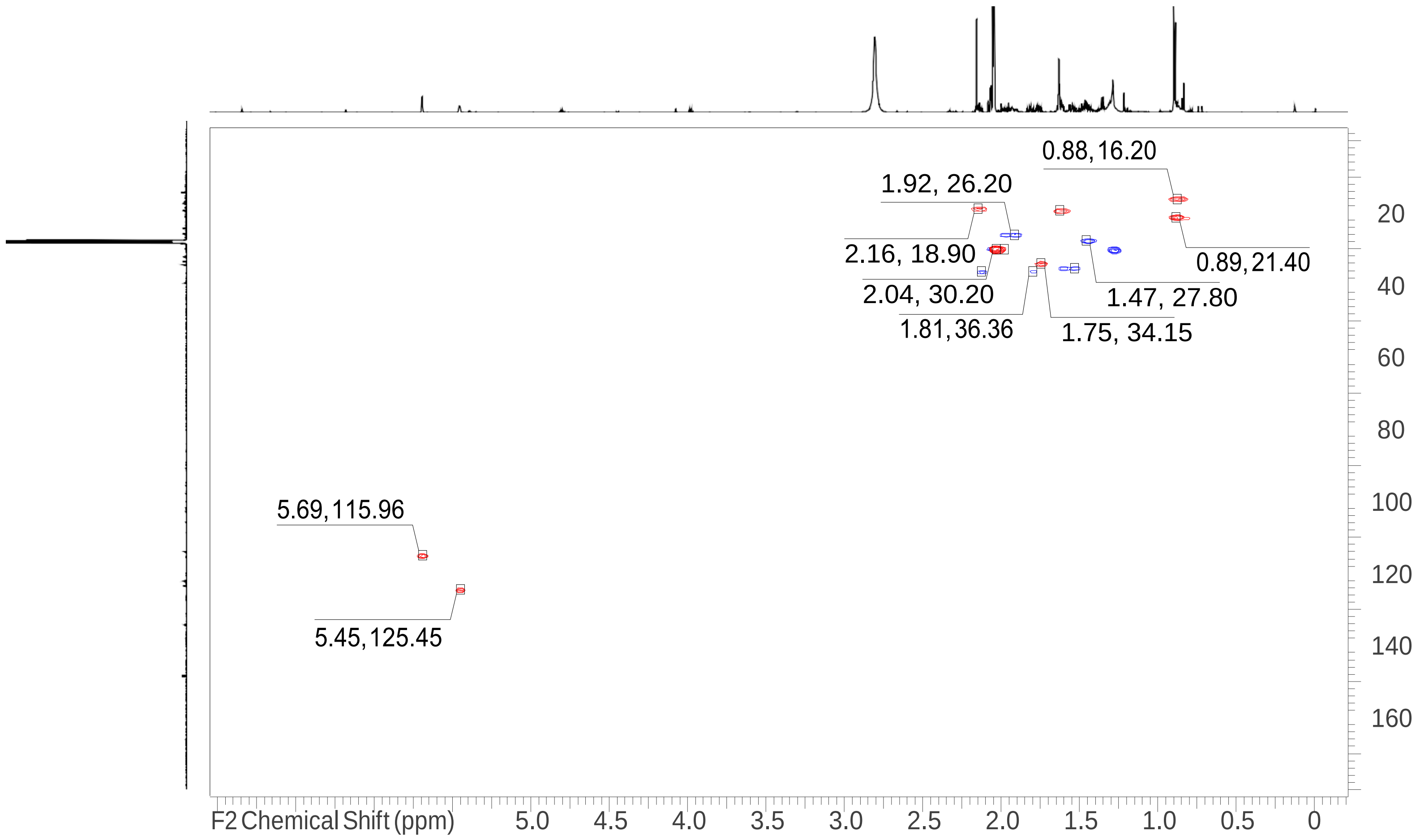

Figure 24: HSQC spectrum in acetone-*d₄* (^{1}H 500 MHz, ^{13}C 125) of **(4).**

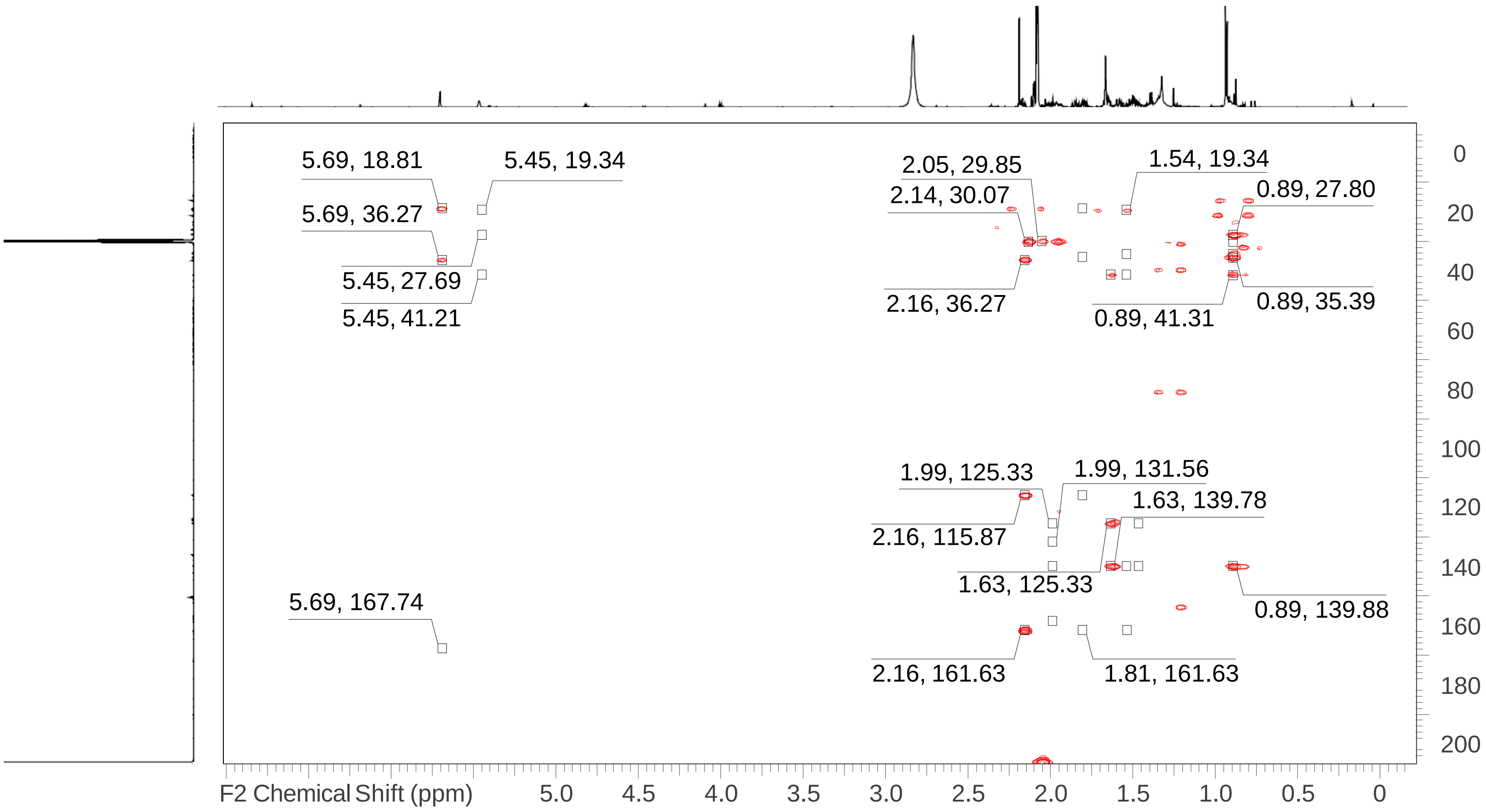

Figure 25: HMBC spectrum in acetone-d_6 (^{1}H 500 MHz, ^{13}C 125 MHz) of (**4**).

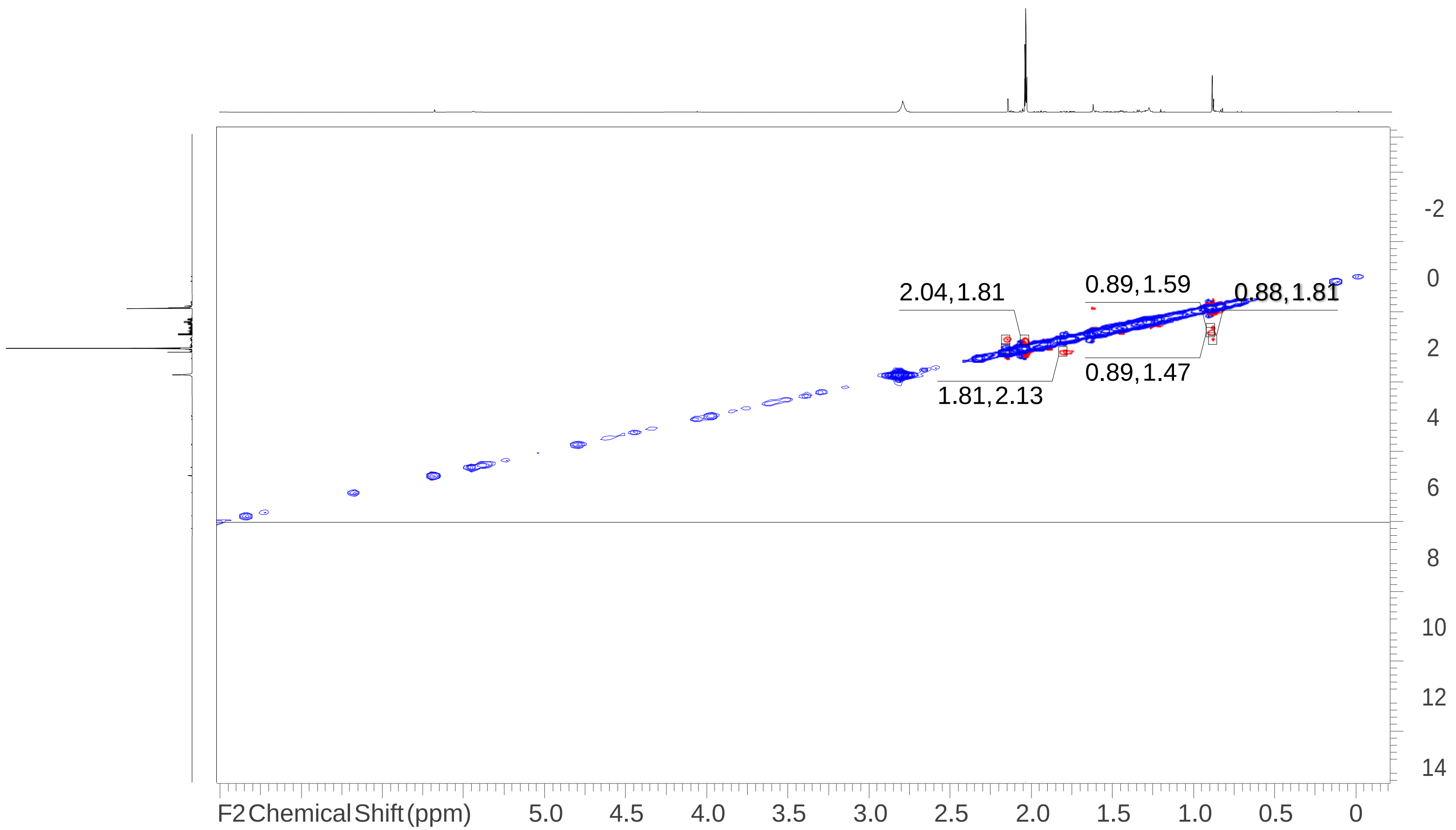

Figure 26: ROESY spectrum in acetone-d_6 (^{1}H 500 MHz) of (**4**).

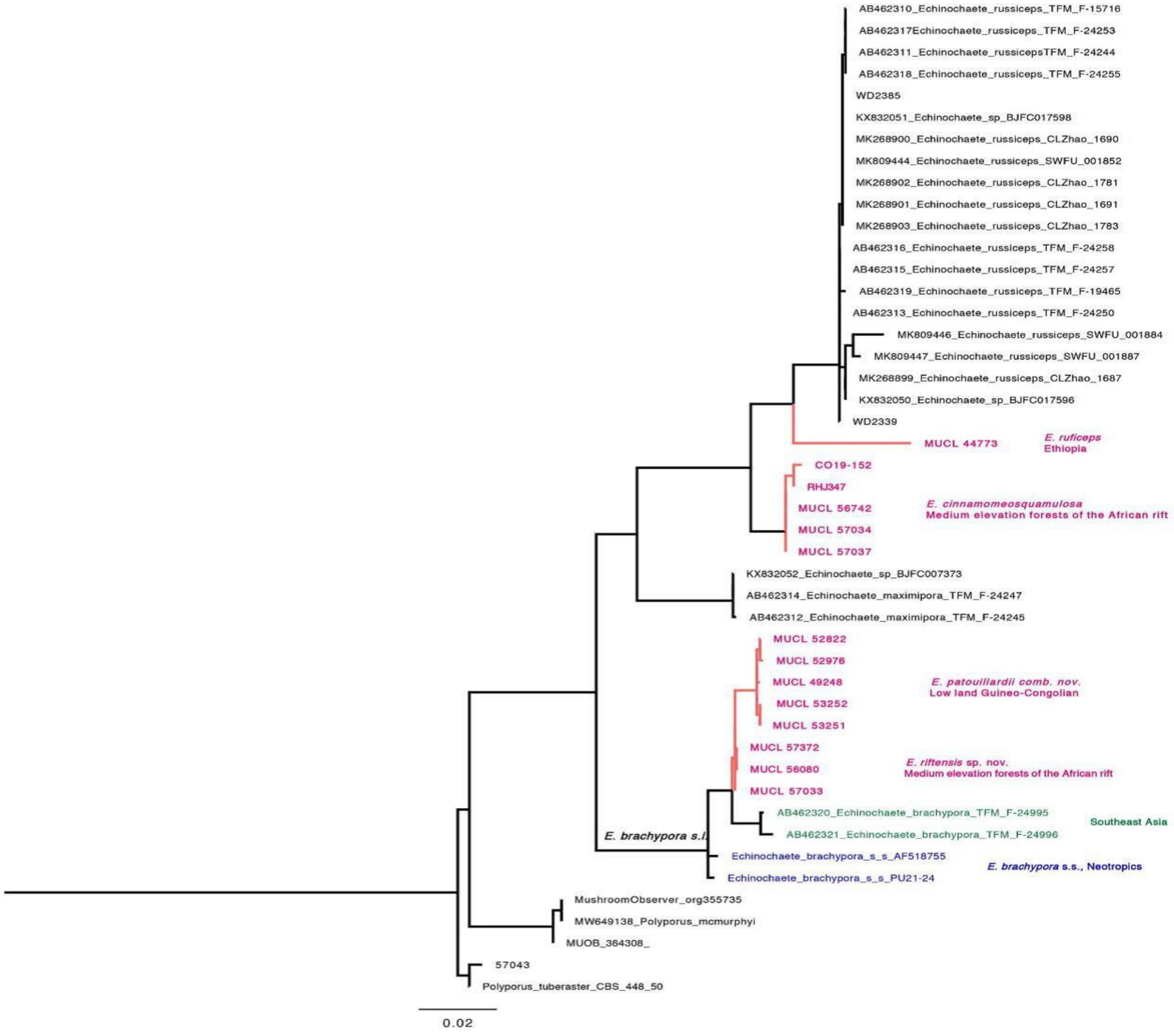

Figure S27: Phylogeny of Echinochaete, based on LSU and ITS.

Table S1: MIC experiment parameters

Test organisms	Strain-Nr.	Growth medium	Incubation temp. [°C]	Positive controls (references)
Escherichia coli	DSM498	MHB [1]	37	Ciproflaxin 1.0 mg/mL
Bacillus subtilis	DSM10	MHB [1]	30	Ciproflaxin 1.0 mg/mL
Mucor plumbeus	MUCL 49355	MYC [2]	30	Nystatin 1.0 mg/mL

[1]MHB: Müller-Hinton Broth (SN X927.1, Carl Roth GmbH, Karlsruhe, Germany); [2]MYC: 1 % w/v, bacto peptone, 1% w/v yeast extract, 2 % w/v glycerol, pH 6.3.

Antimicrobial assay protocol

The assay was conducted as a minimum inhibitory concentration (MIC) assay in 96-well roundbottom microtiter plates using the parameters summarized in Table S38 and as already described in [28-30].

Stocks of the test organisms were generated by growing the organisms overnight in 50 mL shaking flasks filled with 25 mL of the growth medium at 140 rpm (for media and temperatures see Table S13). If the organisms were well grown the next day, which was checked by occurrence of an optical density (OD)>30 of the suspension (OD600 nm for bacteria, OD548 nm for *M. plumbeus*), aliquots of these were stored in 1.5 mL reaction tubes in a freezer at −80 °C for up to 12 months. Upon use, aliquots were unthawed and the OD of the suspension measured and adjusted by diluting with the respective growth medium. OD600 nm was adjusted to 0.01 and OD548 nm to 0.1.

Subsequently, 150 µL of the adjusted suspensions were added to all wells of a 96-well microtiter plate (one test organism per plate). In row A, additional 130 µL of suspensions plus 20 µL of the test compounds (1 mg/mL) and the controls (one compound/column) were added. The test compounds were dissolved in MeOH, MeOH was used as negative control, while different positive controls (references) were used for the test organisms (see Table S13). Then, starting from row A, 150 µL of the suspension were transferred to the next row, the contents thoroughly mixed, and 150 µL transferred to the following row. The remaining 150 µL after row H were discarded. This resulted in a serial dilution of the test compounds, ranging from 66.7 µg/mL in row A to 0.52 µg/mL in row H. The microtiter plates were then incubated overnight on a microplate shaker

at 800 rpm at 30 or 37 °C (see Table S13) and were visually evaluated the next day. The MIC is defined as the lowest concentration were no growth of the test organism was observed. A lower MIC thus corresponds to a higher antimicrobial activity of the test compound.

Becker, K.; Wessel, A.C.; Luangsa-Ard, J.J.; Stadler, M. Viridistratins A-C, antimicrobial and cytotoxic benzo[j]fluoranthenes from stromata of *Annulohypoxylon viridistratum* (hypoxylaceae, ascomycota). *Biomolecules* **2020**, *10*, 1–11, doi:10.3390/biom10050805.

Kemkuignou, B.M.; Treiber, L.; Zeng, H.; Schrey, H.; Schobert, R.; Stadler, M. Macrooxazoles a–d, new 2,5-disubstituted oxazole-4-carboxylic acid derivatives from the plant pathogenic fungus *Phoma macrostoma*. *Molecules* **2020**, *25*, 1–18, doi:10.3390/molecules25235497.

Hassan, K.; Matio Kemkuignou, B.; Stadler, M. Two new triterpenes from basidiomata of the medicinal and edible mushroom, *Laetiporus sulphureus*. *Molecules* **2021**, *26*, 7090, doi:10.3390/molecules26237090.

Sequences of 12 strains from *Genus Echinochaete*

MUCL 56080 16S rRNA sequence

ATAGAATTCCACGTGTAGCAGTGAAATGCGTAGAGATGTGGAGGAATACCGATGGCGAAGGCAGCCCCCTGGGATAACACTGACGCTCATGCA
CGAAAGCGTGGGGAGCAAACAGGATTAGATACCCTGGTAGTCCACGCCCTAAACGATGTCAACTAGTTGTTGGGGATTCATTTCCTTAGTAACG
TAGCTAACGCGTGAAGTTGACCGCCTGGGGAGTACGGTCGCAAGATTAAAACTCAAAGGAATTGACGGGGACCCGCACAAGCGGTGGATGATG
TGGATTAATTCGATGCAACGCGAAAAACCTTACCTACCCTTGACATGCCACTAACGAAGCAGAGATGCATTAGGTGCTCGAAAGAGAAAGTGGA
CACAGGTGCTGCATGGCTGTCGTCAGCTCGTGTCGTGAGATGTTGGGTTAAGTCCCGCAACGAGCGCAACCCTTGTCTCTAGTTGCTACGAAAGG
GCACTCTAGAGAGACTGCCGGTGACAAACCGGAGGAAGGTGGGGATGACGTCAAGTCCTCATGGCCCTTATGGGTAGGGCTTCACACGTCATAC
AATGGTGCATACAGAGGGTTGCCAAGCCGCGAGGTGGAGCCAATCCCAGAAAATGCATCGTAGTCCGGATCGTAGTCTGCAACTCGACTACGTG
AAGCTGGAATCGCTAGTAATCGCGGATCAGCATGCCGCGGTGAATACGTTCCCGGGTCTTGTACACACCGCCCGTCACACCATGGGA

MUCL 56080 16S rRNA sequence BLAST results

Sequences producing significant alignments Download Select columns Show 100

select all *100 sequences selected* GenBank Graphics Distance tree of results MSA Viewer

Description	Scientific Name	Max Score	Total Score	Query Cover	E value	Per. Ident	Acc. Len	Accession
Ralstonia syzygii subsp. indonesiensis strain UQRS 464 16S ribosomal RNA, partial seque…	Ralstonia syzygii subsp. indonesiensis	1367	1367	99%	0.0	99.87%	1400	NR_134150.1
Ralstonia pickettii strain JCM 5969 16S ribosomal RNA, partial sequence	Ralstonia pickettii	1367	1367	99%	0.0	99.87%	1460	NR_113352.1
Ralstonia pickettii strain ATCC 27511 16S ribosomal RNA, partial sequence	Ralstonia pickettii	1367	1367	99%	0.0	99.87%	1491	NR_043152.1
Ralstonia pickettii strain NBRC 102503 16S ribosomal RNA, partial sequence	Ralstonia pickettii	1367	1367	99%	0.0	99.87%	1463	NR_114126.1
Ralstonia insidiosa strain AU2944 16S ribosomal RNA, partial sequence	Ralstonia insidiosa	1367	1367	99%	0.0	99.87%	1436	NR_025242.1
Ralstonia solanacearum strain K60-1 16S ribosomal RNA, partial sequence	Ralstonia solanacearum	1362	1362	99%	0.0	99.73%	1434	NR_118984.1
Ralstonia solanacearum strain K60-1 16S ribosomal RNA, partial sequence	Ralstonia solanacearum	1362	1362	99%	0.0	99.73%	1491	NR_044040.1
Ralstonia syzygii strain ATCC 49543 16S ribosomal RNA, partial sequence	Ralstonia syzygii	1360	1360	99%	0.0	99.60%	1513	NR_040803.1
Ralstonia syzygii strain R001 16S ribosomal RNA, partial sequence	Ralstonia syzygii	1360	1360	99%	0.0	99.73%	1445	NR_025975.1
Ralstonia syzygii subsp. celebesensis strain UQRS 627 16S ribosomal RNA, partial sequence	Ralstonia syzygii subsp. celebesensis	1356	1356	99%	0.0	99.60%	1401	NR_134149.1
Ralstonia mannitolilytica strain LMG 6866 16S ribosomal RNA, partial sequence	Ralstonia mannitolilytica	1356	1356	99%	0.0	99.60%	1449	NR_025385.1
Ralstonia pseudosolanacearum strain UQRS 461 16S ribosomal RNA, partial sequence	Ralstonia pseudosolanacearum	1349	1349	99%	0.0	99.46%	1394	NR_134148.1
Cupriavidus campinensis strain WS2 16S ribosomal RNA, partial sequence	Cupriavidus campinensis	1328	1328	99%	0.0	98.92%	1484	NR_025137.1
Cupriavidus pinatubonensis strain 1245 16S ribosomal RNA, partial sequence	Cupriavidus pinatubonensis	1312	1312	99%	0.0	98.52%	1524	NR_040987.1
Cupriavidus necator strain N-1 16S ribosomal RNA, partial sequence	Cupriavidus necator	1306	1306	99%	0.0	98.38%	1520	NR_102851.1
Cupriavidus numazuensis NBRC 100056 16S ribosomal RNA, partial sequence	Cupriavidus numazuensis NBRC 1000…	1306	1306	99%	0.0	98.38%	1457	NR_113877.1
Cupriavidus lacunae strain S23 16S ribosomal RNA, partial sequence	Cupriavidus lacunae	1303	1303	99%	0.0	98.25%	1468	NR_164934.1
Cupriavidus yeoncheonensis strain DCY86 16S ribosomal RNA, partial sequence	Cupriavidus yeoncheonensis	1303	1303	99%	0.0	98.26%	1449	NR_145541.1
Cupriavidus nantongensis strain X1 16S ribosomal RNA, partial sequence	Cupriavidus nantongensis	1301	1301	99%	0.0	98.25%	1405	NR_149305.1

MUCL 56080 ITS sequence

AGAAGTAAAAGTCGTAACAAGGTTTCCGTAGGTGAACCTGCGGAAGGATCATTAATGAGTCTTGATGCGGGGTTGCAGCTGGTCTTCATAGACACG
TGCTCACCCTGTTCAATCCACTCTACACCTGTGCACTTACTGTGGGTTGCCGGTTGACAGGCAAAGGAGGAGTGCATGTATAATGCATGCCCTTTCT
CTCTGGATCTGGCCTCACGTTTTTATTACACACAAGTATTAGAATGTGTACTGCGATGTTGTAACGCATTTATATACAACTTTCAGCAACGGATCTC
TTGGCTCTCGCATCGATGAAGAACGCAGCGAAATGCGATAAGTAATGTGAATTGCAGAATTCAGTGAATCATCGAATCTTTGAACGCACCTTGCGC
TCCTTGGTATTCCGAGGAGCACGCCTGTTTGAGTGTCATGAAATTCTCAACCTTATATGCCCTTGTTATGGGGGTCTGTAAAGTTGGACTTGGAGGT
GTATTGTCAGCTTGCCCTCTGCGAGTTGGCTCCTCTCAAATGCATTAGCTTTAGTTCCTTTGTGGATCGGCTTTCGGTGTGATAGTTGT

GTCTACGCCGTGACCGTGAAGCGTTTATGGCATAAGCTTATAAACCCCAACGCCTCTTTCCCATTCTTTGGGACAGCGTTCTCTTGACAATCTGACC
TCAAATCAGGTGGGATTACCCGCTGAACTTAAGCAT

Sequences producing significant alignments Download Select columns Show 100

select all 100 sequences selected GenBank Graphics Distance tree of results MSA Viewer

	Description	Scientific Name	Max Score	Total Score	Query Cover	E value	Per. Ident	Acc. Len	Accession
☑	Echinochaete brachypora genes for ITS1, 5.8S rRNA, ITS2, partial and complete sequence, specimen_voucher: T...	Echinochaete br...	883	883	70%	0.0	98.60%	499	AB462320.1
☑	Echinochaete brachypora genes for ITS1, 5.8S rRNA, ITS2, partial and complete sequence, specimen_voucher: T...	Echinochaete br...	867	867	70%	0.0	98.00%	499	AB462321.1
☑	Polyporus udus 5.8S ribosomal RNA gene, partial sequence; internal transcribed spacer 2, complete sequence; a...	Bresadolia uda	647	647	54%	0.0	97.13%	421	AF518755.1
☑	Lentinus squarrosulus isolate SJ06 small subunit ribosomal RNA gene, partial sequence; internal transcribed spac...	Lentinus squarro...	531	531	84%	7e-146	83.31%	668	OK446673.1
☑	Lentinus tigrinus isolate MG331_K4-2D small subunit ribosomal RNA gene, partial sequence; internal transcribed ...	Lentinus tigrinus	523	523	99%	1e-143	81.28%	675	MG208016.1
☑	Polyporus tuberaster voucher KA12-1241 18S ribosomal RNA gene, partial sequence; internal transcribed spacer ...	Polyporus tubera...	518	518	84%	5e-142	83.47%	597	KR673494.1
☑	Uncultured fungus clone LX037984-122-008-H02 internal transcribed spacer 1, partial sequence; 5.8S ribosomal ...	uncultured fungus	516	516	84%	2e-141	82.90%	703	GU053785.1
☑	Amauroderma schomburgkii voucher HUEFS:DHCR504 small subunit ribosomal RNA gene, partial sequence; inte...	Amauroderma sc...	512	512	96%	2e-140	81.30%	694	MF409958.1
☑	Amauroderma schomburgkii voucher ICN202153 small subunit ribosomal RNA gene, partial sequence; internal tra...	Amauroderma sc...	512	512	96%	2e-140	81.30%	695	MW541365.1
☑	Amauroderma schomburgkii voucher ICN202136 small subunit ribosomal RNA gene, partial sequence; internal tra...	Amauroderma sc...	507	507	96%	1e-138	81.22%	693	MW541364.1
☑	Echinochaete sp. voucher BJFC007373 internal transcribed spacer 1, partial sequence; 5.8S ribosomal RNA gene...	Echinochaete sp.	505	505	94%	4e-138	81.67%	660	KX832052.1
☑	Amauroderma schomburgkii voucher URM 84228 internal transcribed spacer 1, partial sequence; 5.8S ribosomal ...	Amauroderma sc...	497	497	96%	7e-136	81.02%	643	KC348457.1
☑	Polyporus guianensis CulTENN10064 18S ribosomal RNA gene, partial sequence; internal transcribed spacer 1, 5...	Polyporus guiane...	497	497	99%	7e-136	80.38%	1056	AF516565.1
☑	Ganoderma sp. BAB-4725 18S ribosomal RNA gene, partial sequence; internal transcribed spacer 1, 5.8S riboso...	Ganoderma sp. ...	496	496	97%	2e-135	80.88%	675	KR154954.1
☑	Amauroderma schomburgkii voucher URM 84226 internal transcribed spacer 1, partial sequence; 5.8S ribosomal ...	Amauroderma sc...	494	494	96%	9e-135	80.90%	649	KC348456.1
[illegible]	[illegible]	[illegible]	[illegible]	[illegible]	[illegible]	[illegible]	[illegible]	[illegible]	[illegible]

MUCL 56080 LSU sequence

GGAGAAGTGCTTTCTGTGCTGGACCGTGTATAAGTCTCTTGGAATAGAGCGTCATAGAGGGTGAGAATCCCGTCTTTGACACGGACTACCAGTGCT
CTGTGATGCGCTCTCAAAGAGTCGAGTTGTTTGGGAATGCAGCTCAAAATGGGTGGTAAATTCCATCTAAAGCTAAATATTGGCGAGAGACCGATA
GCGAACAAGTACCGTGAGGGAAAGATGAAAAGCACTTTGGAAAGAGAGTTAAACAGTACGTGAAATTGCTGAAAGGGAAACGCTTGAAGTCAGT
CGCGTTGTTTGGAACTCAGCCTTGCTCTTTGCTTGGTGCACTTTCTGAATGACGGGCCAGCATCGATTTTGACCGTTGGAAAAGGGCTGGAGGAATG
TGGCACCTTTGGGTGTGTTATAGCCTCCTGTCGCATACAGCGGTTGGGATCGAGGAACGCAGCGCGCCGCAAGGCAGGGGTTCGCCCACTTTCGCG
CTTAGGATGCTGGCATAATGGCTTTAAACGACCCGTCTTGAAACACGGACCAAGGAGTCTAACATACCTGCGAGTGTTTGGGTGGAAAACCCGAGC
GCGTAATGAAAGTGAAAGTTGAGACCTCTGTCGTGGAGGGCATCGACGCCCGGACCTGACGTTCTCTGAAGGATCCGCGGTGGAGCATGTATGTTG
GGACCCGAAAGATGGTGAACTATGCCTGAATAGGGTGAAGCCAGAGGAAACTCTGGTGGAGGCTCGTAGCGATTCTGACGTGCAAATCGATCGTC
AAATTTGGGTATAGGGGCGAAAGACTAATCGAACCATCTAGTAGCTGGTTCCTGCCGAAGTTTCCCTCAGGATAGCAGAAACTCATTATCAGATTT
ATGtGGTAAAGCGAATGATTAGAGGCCTTGGGGTTGAAACAACCTTAACCTATTCTCAAACTTTAAATATGTAAGAACGAGCCGTCGCTTGATTGG
ACCGCTCGGCGATTGAGAGTTTCTAGTGGGCCATTTTTGGTAAGCAGAACTGGCGATGCGGGATGAACCGAACGCGAGGTTAAGGTGCCGGAATA
CACGCTCATCAGACACCACAAAAGGTGTTAGTTCATCTAGACAGCAGGACGGTGGCCATGGAAGTCGGAATCCGCTAAGGAGTGTGTAACAACTC

ACCTGCCGAATGAACTAGCCCTGAAAATGGATGGCGCTCAAGCGTGTTACCCA

Sequences producing significant alignments Download Select columns Show 100

select all *100 sequences selected* GenBank Graphics Distance tree of results MSA Viewer

	Description	Scientific Name	Max Score	Total Score	Query Cover	E value	Per. Ident	Acc. Len	Accession
☑	Echinochaete sp. voucher BJFC007373 28S ribosomal RNA gene, partial sequence	Echinochaete sp.	2156	2156	100%	0.0	99.08%	1385	KX832061.1
☑	Megasporia sp. voucher Cui 11144 28S ribosomal RNA gene, partial sequence	Megasporia sp.	2111	2111	100%	0.0	98.42%	1381	KX900697.1
☑	Polyporus sp. voucher CTR-3-23 large subunit ribosomal RNA gene, partial sequence	Polyporus sp.	2111	2111	100%	0.0	98.42%	1308	MT950182.1
☑	Polyporus sp. voucher CTR-2-49 large subunit ribosomal RNA gene, partial sequence	Polyporus sp.	2111	2111	100%	0.0	98.42%	1345	MT950168.1
☑	Polyporus sp. voucher CTR-1-6.1 large subunit ribosomal RNA gene, partial sequence	Polyporus sp.	2111	2111	100%	0.0	98.42%	1350	MT950166.1
☑	Polyporus sp. voucher CT-16F large subunit ribosomal RNA gene, partial sequence	Polyporus sp.	2109	2109	100%	0.0	98.33%	1354	MT950177.1
☑	Polyporus sp. ZJL-20164 voucher Dai 13950 25S large subunit ribosomal RNA gene, partial sequence	Polyporus sp. ZJ...	2108	2108	100%	0.0	98.33%	1343	KX900170.1
☑	Polyporus sp. ZJL-20164 voucher Cui 6870 25S large subunit ribosomal RNA gene, partial sequence	Polyporus sp. ZJ...	2106	2106	100%	0.0	98.33%	1343	KX900167.1
☑	Polyporus sp. ZJL-20164 voucher Yuan 6639 25S large subunit ribosomal RNA gene, partial sequence	Polyporus sp. ZJ...	2106	2106	100%	0.0	98.33%	1343	KX900165.1
☑	Abundisporus pubertatis voucher Dai 11927 25S large subunit ribosomal RNA gene, partial sequence	Abundisporus pu...	2106	2106	100%	0.0	98.33%	1351	KC867494.1
☑	Abundisporus pubertatis voucher Dai 7254 28S large subunit ribosomal RNA gene, partial sequence	Abundisporus pu...	2106	2106	100%	0.0	98.33%	1378	KC787577.1
☑	Abundisporus pubertatis voucher Dai 11310 28S large subunit ribosomal RNA gene, partial sequence	Abundisporus pu...	2106	2106	100%	0.0	98.33%	1379	KC787575.1
☑	Abundisporus pubertatis voucher Cui 8607 28S large subunit ribosomal RNA gene, partial sequence	Abundisporus pu...	2106	2106	100%	0.0	98.33%	1380	KC787574.1
☑	Polyporus sp. voucher CTR-2-23 large subunit ribosomal RNA gene, partial sequence	Polyporus sp.	2106	2106	100%	0.0	98.33%	1353	MT950180.1
☑	Polyporus sp. voucher CTR-1-7.2 large subunit ribosomal RNA gene, partial sequence	Polyporus sp.	2106	2106	100%	0.0	98.33%	1351	MT950178.1

MUCL 52822 16S rRNA sequence

AAGCGTTAATCGGAATTACTGGGCGTAAAGCGTGCGCAGGCGGTTGTGCAAGACCGATGTGAAATCCCCGGGCTTAACCTGGGAATTGCATT
GGTGACTGCACGGCTAGAGTGTGTCAGAGGGGGGTAGAATTCCACGTGTAGCAGTGAAATGCGTAGAGATGTGGAGGAATACCGATGGCGAA
GGCAGCCCCCTGGGATAACACTGACGCTCATGCACGAAAGCGTGGGGAGCAAACAGGATTAGATACCCTGGTAGTCCACGCCCTAAACGATG
TCAACTAGTTGTTGGGGATTCATTTCCTTAGTAACGTAGCTAACGCGTGAAGTTGACCGCCTGGGGAGTACGGTCGCAAGATTAAAACTCAAA
GGAATTGACGGGGACCCGCACAAGCGGTGGATGATGTGGATTAATTCGATGCAACGCGAAAAACCTTACCTACCCTTGACATGCCACTAACG
AAGCAGAGATGCATTAGGTGCTCGAAAGAGAAAGTGGACACAGGTGCTGCATGGCTGTCGTCAGCTCGTGTCGTGAGATGTTGGGTTAAGTC
CCGCAACGAGCGCAACCCTTGTCTCTAGTTGCTACGAAAGGGCACTCTAGAGAGACTGCCGGTGACAAACCGGAGGAAGGTGGGGATGACGT
CAAGTCCTCATGGCCCTTATGGGTAGGGCTTCACACGTCATACAATGGTGCATACAGAGGGTTGCCAAGCCGCGAGGTGGAGCCAATCCCAG
AAAATGCATCGTAGTCCGGATCGTAGTCTGCAACTCGACTACGTGAAGCTGGAATCGCTAGTAATCGCGGATCAGCATGCCGCGGTGAATAC
GTTCCCGGGTCTTGTACACACCGCCCGTCACACCATGG

MUCL 52976 16S rRNA sequence

GTCCGGGAAGAAATCGCACCTGATAATACCGGGTGTGGATGACGGTACCGGAAGAATAAGGACCGGCTAACTACGTGCCAGCAGCCGCGGTA
ATACGTAGGGTCCAAGCGTTAATCGGAATTACTGGGCGTaAAAGCGTGCGCAGGCtGGTTGTGCAAGACCGATGTGAAATCCCCGGGCTTAAC
CTGGGAATTGCATTGGTGACTGCACGGCTAGAGTGTGTCAGAGGGGGGTAGAATTCCACGTGTAGCAGTGAAATGCGTAGAGATGTGGAGGA
ATACCGATGGCGAAGGCAGCCCCCTGGGATAACACTGACGCTCATGCACGAAAGCGTGGGGAGCAAACAGGATTAGATACCCTGGTAGTCCA
CGCCCTAAACGATGTCAACTAGTTGTTGGGGATTCATTTCCTTAGTAACGTAGCTAACGCGTGAAGTTGACCGCCTGGGGAGTACGGTCGCAA
GATTAAAACTCAAAGGAATTGACGGGGACCCGCACAAGCGGTGGATGATGTGGATTAATTCGATGCAACGCGAAAAACCTTACCTACCCTTG
ACATGCCACTAACGAAGCAGAGATGCATTAGGTGCTCGAAAGAGAAAGTGGACACAGGTGCTGCATGGCTGTCGTCAGCTCGTGTCGTGAGA
TGTTGGGTTAAGTCCCGCAACGAGCGCAACCCTTGTCTCTAGTTGCTACGAAAGGGCACTCTAGAGAGACTGCCGGTGACAAACCGGAGGAA
GGTGGGGATGACGTCAAGTCCTCATGGCCCTTATGGGTAGGGCTTCACACGTCATACAATGGTGCATACAGAGGGTTGCCAAGCCGCGAGGT
GGAGCCAATCCCAGAAAATGCATCGTAGTCCGGATCGTAGTCTGCAACTCGACTACGTGAAGCTGGAATCGCTAGTAATCGCGGATCAGCAT
GCCGCGGTGAATACGTTCCCGGGTCTTGTACACACCGCCCGTCACACCATGGGAGTGGGTTTTGCCAGAAGTAGTTAGCCTAACCGCAAGGGG
GCGATTACCACGGCAGGGTTCATGACTGGGGTGAAGTCGTACAGGGATAA

MUCL 53251 16S rRNA sequence

GCAGGCGGTTGTGCAAGACCGATGTGAAATCCCCGGGCTTAACCTGGGAATTGCATTGGTGACTGCACGGCTAGAGTGTGTCAGAGGGGGGT
AGAATTCCACGTGTAGCAGTGAAATGCGTAGAGATGTGGAGGAATACCGATGGCGAAGGCAGCCCCCTGGGATAACACTGACGCTCATGCAC
GAAAGCGTGGGGAGCAAACAGGATTAGATACCCTGGTAGTCCACGCCCTAAACGATGTCAACTAGTTGTTGGGGATTCATTTCCTTAGTAACG
TAGCTAACGCGTGAAGTTGACCGCCTGGGGAGTACGGTCGCAAGATTAAAACTCAAAGGAATTGACGGGGACCCGCACAAGCGGTGGATGAT
GTGGATTAATTCGATGCAACGCGAAAAACCTTACCTACCCTTGACATGCCACTAACGAAGCAGAGATGCATTAGGTGCTCGAAAGAGAAAGT
GGACACAGGTGCTGCATGGCTGTCGTCAGCTCGTGTCGTGAGATGTTGGGTTAAGTCCCGCAACGAGCGCAACCCTTGTCTCTAGTTGCTACG
AAAGGGCACTCTAGAGAGACTGCCGGTGACAAACCGGAGGAAGGTGGGGATGACGTCAAGTCCTCATGGCCCTTATGGGTAGGGCTTCACAC
GTCATACAATGGTGCATACAGAGGGTTGCCAAGCCGCGAGGTGGAGCCAATCCCAGAAAATGCATCGTAGTCCGGATCGTAGTCTGCAACTC

GACTACGTGAAGCTGGAATCGCTAGTAATCGCGGATCAGCATGCCGCGGTGAATACGTTCCCGGGTCTTGTACACACCGCCCGTCACACCATG
GGAGTGGGTTTTGCCAGAAGTAGTTAGCCTAACCGCAAGGGGGGCGATTACCACGGCAGGGTTCATGACTGGGGTGAAGTCTACAGAGG

MUCL 53252 16S rRNA sequence

GTAAAGGCCTACCAAGGCGACGATCASTAGCTGGTCTGAGAGGACGATCAGCCACACTGGGACTGAGACACGGCCCAGACTCCTACGGGAGG
CAGCAGTGGGGAATTTTGGACAATGGSGGCAACCCTGATCCAGCAATGCCGCGTGTGTGAAGAAGGCCTTCGGGTTGTAAAGCACTTTTGTC
CGGGAAGAAATCGCACCTGATAATACCGGGWGTGGATGACGGTACCGGAAGAATAAGGACCGGCTAACTACGTGCCAGCAGCCGCGGTAAT
ACGTAGGGTCCAAGCGTTAATCGGAATTACTGGGCGTAAAGCGTGCGCAGGCGGTTGTGCAAGACCGATGTGAAATCCCCGGGCTTAACCTG
GGAATTGCATTGGTGACTGCACGGCTAGAGTGTGTCAGAGGGGGGTAGAATTCCACGTGTAGCAGTGAAATGCGTAGAGATGTGGAGGAATA
CCGATGGCGAAGGCAGCCCCCTGGGATAACACTGACGCTCATGCACGAAAGCGTGGGGAGCAAACAGGATTAGATACCCTGGTAGTCCACGC
CCTAAACGATGTCAACTAGTTGTTGGGGATTCATTTCCTTAGTAACGTAGCTAACGCGTGAAGTTGACCGCCTGGGGAGTACGGTCGCAAGAT
TAAAACTCAAAGGAATTGACGGGGACCCGCACAAGCGGTGGATGATGTGGATTAATTCGATGCAACGCGAAAAAACCTTACCTACCCTTGAC
ATGCCACTAACGAAGCAGAAGATGCATTAGGTGCTCGAAAGAGAAAGTGGACACAGGTGCTGCATGGCTGTCGTCAGCTCGTGTCGTGAGAT
GTTGGGTTAAGTCCCGCAACGAGCGCAACCCTTGTCTCTAGTTGCTACGAAAGGGCACTCTAGAGAGACTGCCGGTGACAAACCGGAGGAAG
GTGGGGATGACGTCAAGTCCTCATGGCCCTTATGGGTAGGGCTTCACACGTCATACAATGGTGCATACAGAGGGTTGCCAAGCCGCGAGGTG
GAGCCAATCCCAGAAAAATGCATCGTAGTCCGGATCGTAGTCTGCAACTCGACTACGTGAAGCTGGAATCGCTAGTAATCGCGGATCAGCAT
GCCGCGGTGAATACGTTCCCGGGTCTTGTACACACCGCCCGTCACACCATGGGAGTGGGTTTTGCCAGAAGTAGTTAGCCTAACCGCAAGGAG
GGCGATTACCACGGCAGGGTTCATGACTGG

MUCL 57033 16S rRNA sequence

TAGAATTCCACGTGTAGCAGTGAAATGCGTAGAGATGTGGAGGAATACCGATGGCGAAGGCAGCCCCCTGGGATAACACTGACGCTCATGCA
CGAAAGCGTGGGGAGCAAACAGGATTAGATACCCTGGTAGTCCACGCCCTAAACGATGTCAACTAGTTGTTGGGGATTCATTTCCTTAGTAAC
GTAGCTAACGCGTGAAGTTGACCGCCTGGGGAGTACGGTCGCAAGATTAAAACTCAAAGGAATTGACGGGGACCCGCACAAGCGGTGGATG
ATGTGGATTAATTCGATGCAACGCGAAAAACCTTACCTACCCTTGACATGCCACTAACGAAGCAGAGATGCATTAGGTGCTCGAAAGAGAAA
GTGGACACAGGTGCTGCATGGCTGTCGTCAGCTCGTGTCGTGAGATGTTGGGTTAAGTCCCGCAACGAGCGCAACCCTTGTCTCTAGTTGCTA
CGAAAGGGCACTCTAGAGAGACTGCCGGTGACAAACCGGAGGAAGGTGGGGATGACGTCAAGTCCTCATGGCCCTTATGGGTAGGGCTTCAC
ACGTCATACAATGGTGCATACAGAGGGTTGCCAAGCCGCGAGGTGGAGCCAATCCCAGAAAATGCATCGTAGTCCGGATCGTAGTCTGCAAC
TCGACTACGTGAAGCTGGAATCGCTAGTAATCGCGGATCAGCATGCCGCGGTGAATACGTTCCCGGGTCTTGTACACACCGCCCGTCACACCA
TGGGAGTGGGTTTTGCCAGAAGTAGTTAGCCTAACCGCAAG

MUCL 57034 16S rRNA sequence

GTACCGGAAGAATAAGGACCGGCTAACTACGTGCCAGCAGCCGCGGTAATACGTAGGGTCCAAGCGTTAATCGGAATTACTGGGCGTAAAGC
GTGCGCAGGCGGTTGTGCAAGACCGATGTGAAATCCCCGGGCTTAACCTGGGAATTGCATTGGTGACTGCACGGCTAGAGTGTGTCAGAGGG
GGGTAGAATTCCACGTGTAGCAGTGAAATGCGTAGAGATGTGGAGGAATACCGATGGCGAAGGCAGCCCCCTGGGATAACACTGACGCTCAT
GCACGAAAGCGTGGGGAGCAAACAGGATTAGATACCCTGGTAGTCCACGCCCTAAACGATGTCAACTAGTTGTTGGGGATTCATTTCCTTAGT

AACGTAGCTAACGCGTGAAGTTGACCGCCTGGGGAGTACGGTCGCAAGATTAAAACTCAAAGGAATTGACGGGGACCCGCACAAGCGGTGG
ATGATGTGGATTAATTCGATGCAACGCGAAAAACCTTACCTACCCTTGACATGCCACTAACGAAGCAGAGATGCATTAGGTGCTCGAAAGAG
AAAGTGGACACAGGTGCTGCATGGCTGTCGTCAGCTCGTGTCGTGAGATGTTGGGTTAAGTCCCGCAACGAGCGCAACCCTTGTCTCTAGTTG
CTACGAAAGGGCACTCTAGAGAGACTGCCGGTGACAAACCGGAGGAAGGTGGGGATGACGTCAAGTCCTCATGGCCCTTATGGGTAGGGCTT
CACACGTCATACAATGGTGCATACAGAGGGTTGCCAAGCCGCGAGGTGGAGCCAATCCCAGAAAATGCATCGTAGTCCGGATCGTAGTCTGC
AACTCGACTACGTGAAGCTGGAATCGCTAGTAATCGCGGATCAGCATGCCGCGGTGAATACGTTCCCGGGTCTTGTACACACCGCCCGTCACA
CCATGGGAGTGGGTTTTGCCAGAAGTAGTTAGCCTAACCGCAAGGGGGGCGATTACCACGGCAGGGTTCATGACT

MUCL 57037 16S rRNA sequence

AGGCAGCAGTGGGGAATTTTGGACAAGGGGGGCAACCCTGATCCAGCAATGCCGCGTGTGTGAAGAAGGCCTTCGGGTTGTAAAGCACTTTT
GTCCGGGAAGAAATCGCACCTGATAATACCGGGTGTGGATGACGGTACCGGAAGAATAAGGACCGGCTAACTACGTGTCAGCAGCCGCGGTA
ATACGTAGGGTCCAAGCGTTAATCGGAATTACTGGGCGTAAAGCGTGCGCAGGCGGTTGTGCAAGACCGATGTGAAATCCCCGGGCTTAACC
TGGGAATTGCATTGGTGACTGCACGGCTAGAGTGTGTCAGAGGGGGGTAGAATTCCACGTGTAGCAGTGAAATGCGTAGAGATGTGGAGGAA
TACCGATGGCGAAGGCAGCCCCCTGGGATAACACTGACGCTCATGCACGAAAGCGTGGGGAGCAAACAGGATTAGATACCCTGGTAGTCCAC
GCCCTAAACGATGTCAACTAGTTGTTGGGGATTCATTTCCTTAGTAACGTAGCTAACGCGTGAAGTTGACCGCCTGGGGAGTACGGTCGCAAG
ATTAAAACTCAAAGGAATTGACGGGGACCCGCACAAGCGGTGGATGATGTGGATTAATTCGATGCAACGCGAAAAACCTTACCTACCCTTGA
CATGCCACTAACGAAGCAGAGATGCATTAGGTGCTCGAAAGAGAAAGTGGACACAGGTGCTGCATGGCTGTCGTCAGCTCGTGTCGTGAGAT
GTTGGGTTAAGTCCCGCAACGAGCGCAACCCTTGTCTCTAGTTGCTACGAAAGGGCACTCTAGAGAGACTGCCGGTGACAAACCGGAGGAAG
GTGGGGATGACGTCAAGTCCTCATGGCCCTTATGGGTAGGGCTTCACACGTCATACAATGGTGCATACAGAGGGTTGCCAAGCCGCGAGGTG
GAGCCAATCCCAGAAAATGCATCGTAGTCCGGATCGTAGTCTGCAACTCGACTACGTGAAGCTGGAATCGCTAGTAATCGCGGATCAGCATG
CCGCGGTGAATACGTTCCCGGGTCTTGTACACACCGCCCGTCACACCATGGGAGTGGGTTTTGCCAGAAGTAGTTAGCCTAACCGCAAGGAGG
GCGATTACCACGG

MUCL 49248 16S rRNA sequence

ATGCAAGTCGAACGGCAGCACGGGAGCAATCCTGGTGGCGAGTGGCGAACGGGTGAGTAATACATCGGAACGTGCCCTGTAGTGGGGGATA
ACTAGTCGAAAGACTAGCTAATACCGCATACGACCTGAGGGTGAAAGTGGGGGACCGCAAGGCCTCATGCTATAGGAGCGGCCGATGTCTGA
TTAGCTAGTTGGTGGGGTAAAGGCCTACCAAGGCGACGATCAGTAGCTGGTCTGAGAGGACGATCAGCCACACTGGGACTGAGACACGGCCC
AGACTCCTACGGGAGGCAGCAGTGGGGAATTTTGGACAATGGGGGCAACCCTGATCCAGCAATGCCGCGTGTGTGAAGAAGGCCTTCGGGTT
GTAAAGCACTTTTGTCCGGGAAGAAATCGCACCTGATAATACCGGGTGTGGATGACGGTACCGGAAGAATAAGGACCGGCTAACTACGTGCC
AGCAGCCGCGGTAATACGTAGGGTCCAAGCGTTAATCGGAATTACTGGGCGTAAAGCGTGCGCAGGCGGTTGTGCAAGACCGATGTGAAATC
CCCGGGCTTAACCTGGGAATTGCATTGGTGACTGCACGGCTAGAGTGTGTCAGAGGGGGGTAGAATTCCACGTGTAGCAGTGAAATGCGTAG
AGATGTGGAGGAATACCGATGGCGAAGGCAGCCCCCTGGGATAACACTGACGCTCATGCACGAAAGCGTGGGGAGCAAACAGGATTAGATA
CCCTGGTAGTCCACGCCCTAAACGATGTCAACTAGTTGTTGGGGATTCATTTCCTTAGTAACGTAGCTAACGCGTGAAGTTGACCGCCTGGGG
AGTACGGTCGCAAGATTAAAACTCAAAGGAATTGACGGGGACCCGCACAAGCGGTGGATGATGTGGATTAATTCGATGCAACGCGAAAAACC
TTACCTACCCTTGACATGCCACTAACGAAGCAGAGATGCATTAGGTGCTCGAAAGAGAAAGTGGACACAGGTGCTGCATGGCTGTCGTCAGC
TCGTGTCGTGAGATGTTGGGTTAAGTCCCGCAACGAGCGCAACCCTTGTCTCTAGTTGCTACGAAAGGGCACTCTAGAGAGACTGCCGGTGAC
AAACCGGAGGAAGGTGGGGATGACGTCAAGTCCTCATGGCCCTTATGGGTAGGGCTTCACACGTCATACAATGGTGCATACAGAGGGTTGCC
AAGCCGCGAGGTGGAGCCAATCCCAGAAAATGCATCGTAGTCCGGATCGTAGTCTGCAACTCGACTACGTGAAGCTGGAATCGCTAGTAATC
GCGGATCAGCATGCCGCGGTGAATACGTTCCCGGGTCTTGTACACACCGCCCGTCACACCATGGGAGTGGG

MUCL 56742 16S rRNA sequence

ATGCCGCGTGTGTGAAGAAAGGCCTTCGGGTTGTAAAGCACTTTCTGTCCGGGAAGAAATCGCACCTGATAATACCGGGTGTGGATGACGGTA
CCGGAAGAATAAGGACCGGCTAACTACGTGCCAGCAGCCGCGGTAATACGTAGGGTCCAAGCGTTAATCGGAATTACTGGGCGTAAAGCGTG
CGCAGGCGGTTGTGCAAGACCGATGTGAAATCCCCGGGCTTAACCTGGGAATTGCATTGGTGACTGCACGGCTAGAGTGTGTCAGAGGGGGG
TAGAATTCCACGTGTAGCAGTGAAATGCGTAGAGATGTGGAGGAATACCGATGGCGAAGGCAGCCCCCTGGGATAACACTGACGCTCATGCA
CGAAAGCGTGGGGAGCAAACAGGATTAGATACCCTGGTAGTCCACGCCCTAAACGATGTCAACTAGTTGTTGGGGGATTCATTTCCCTTAGTA
AACGTAGCTAACGCGTTGAAGGTTGACCGCCCTGGGGGAGTACGGTTCGCAAGATTTAAAACTCAAAAGGAAATTGACGGGGACCCGCACAA
GCGGTGGATGATGTGGATTAATTCGATGCAACGCGAAAAACCTTACCTACCCTTGACATGCCACTAACGAAGCAGAGATGCATTAGGTGCTCG
AAAGAGAAAGTGGACACAGGTGCTGCATGGCTGTCGTCAGCTCGTGTCGTGAGATGTTGGGTTAAGTCCCGCAACGAGCGCAACCCTTGTCTC
TAGTTGCTACGAAAGGGCACTCTAGAGAGACTGCCGGTGACAAACCGGAGGAAGGTGGGGATGACGTCAAGTCCTCATGGCCCTTATGGGTA
GGGCTTCACACGTCATACAATGGTGCATACAGAGGGTTGCCAAGCCGCGAGGTGGAGCCAATCCCAGAAAATGCATCGTAGTCCGGATCGTA
GTCTGCAACTCGACTACGTGAAGCTGGAATCGCTAGTAATCGCGGATCAGCATGCCGCGGTGAATACGTTCCCGGGTCTTGTACACACCGCCC
GTCACACCATGGGAGTGGGTTTTGCCAGAAGTAGTTAGCCTAACCGCAAGGAGGGCGATTACCACGGCAGGGTTCATG

MUCL 52977 16S rRNA sequence

GGAGCGGCCGATGTCTGATTAGCTAGTTGGTGGGGTAAAGGCCTACCAMGGCGACGATCAGTAGCTGGTCTGAGASGACGATCAGCCACACT
GGRACTGAGACWCGGCCCAGACTCCTACGGGAGGCAGCAGTGGSGAATTTTGGACWATGGGGGCAACCCTGATCCAGCAATGCCGCGTGTG
TGAAGAAGGCCTTCGGGTTGTAAAGCACTTTTGTCCGGGAAGAAATCGCACCTGATAATACCGGGTGTGGATGACGGTACCGGAAGAATAAG

GACCGGCTAACTACGTGCCAGCAGCCGCGGTAATACGTAGGGTCCAAGCGTTAATCGGAATTACTGGGCGTAAAGCGTGCGCAGGCGGTTGT
GCAAGACCGATGTGAAATCCCCGGGCTTAACCTGGGAATTGCATTGGTGACTGCACGGCTAGAGTGTGTCAGAGGGGGGTAGAATTCCACGT
GTAGCAGTGAAATGCGTAGAGATGTGGAGGAATACCGATGGCGAAGGCAGCCCCCTGGGATAACACTGACGCTCATGCACGAAAGCGTGGG
GAGCAAACAGGATTAGATACCCTGGTAGTCCACGCCCTAAACGATGTCAACTAGTTGTTGGGGATTCATTTCCTTAGTAACGTAGCTAACGCG
TGAAGTTGACCGCCTGGGGAGTACGGTCGCAAGATTAAAACTCAAAGGAATTGACGGGGACCCGCACAAGCGGTGGATGATGTGGATTAATT
CGATGCAACGCGAAAAACCTTACCTACCCTTGACATGCCACTAACGAAGCAGAGATGCATTAGGTGCTCGAAAGAGAAAGTGGACACAGGTG
CTGCATGGCTGTCGTCAGCTCGTGTCGTGAGATGTTGGGTTAAGTCCCGCAACGAGCGCAACCCTTGTCTCTAGTTGCTACGAAAGGGCACTC
TAGAGAGACTGCCGGTGACAAACCGGAGGAAGGTGGGGATGACGTCAAGTCCTCATGGCCCTTATGGGTAGGGCTTCACACGTCATACAATG
GTGCATACAGAGGGTTGCCAAGCCGCGAGGTGGAGCCAATCCCAGAAAATGCATCGTAGTCCGGATCGTAGTCTGCAACTCGACTACGTGAA
GCTGGAATCGCTAGTAATCGCGGATCAGCATGCCGCGGTGAATACGTTCCCGGGTCTTGTACACACCGCCCGTCACACCATGGGAGTGGGTTT
TGCCAGAAGTAGTTAGCCTAA

MUCL 57043 16S rRNA sequence

GAGGCAGCAGTGGGGAATTTTGGACAATGGGGgCAACCCTGATCCAGCAATGCCGCGTGTGTGAAGAAGGCCTTCGGGTTGTAAAGCACTTTT
GTCCGGGAAGAAATCGCACCTGATAATACCGGGTGTGGATGACGGTACCGGAAGAATAAGGACCGGCTAACTACGTGCCAGCAGCCGCGGTA
ATACGTAGGGTCCAAGCGTTAATCGGAATTACTGGGCGTAAAGCGTGCGCAGGCGGTTGTGCAAGACCGATGTGAAATCCCCGGGCTTAACC
TGGGAATTGCATTGGTGACTGCACGGCTAGAGTGTGTCAGAGGGGGGTAGAATTCCACGTGTAGCAGTGAAATGCGTAGAGATGTGGAGGAA
TACCGATGGCGAAGGCAGCCCCCTGGGATAACACTGACGCTCATGCACGAAAGCGTGGGGAGCAAACAGGATTAGATACCCTGGTAGTCCAC
GCCCTAAACGATGTCAACTAGTTGTTGGGGATTCATTTCCTTAGTAACGTAGCTAACGCGTGAAGTTGACCGCCTGGGGAGTACGGTCGCAAG
ATTAAAACTCAAAGGAATTGACGGGGACCCGCACAAGCGGTGGATGATGTGGATTAATTCGATGCAACGCGAAAAACCTTACCTACCCTTGA
CATGCCACTAACGAAGCAGAGATGCATTAGGTGCTCGAAAGAGAAAGTGGACACAGGTGCTGCATGGCTGTCGTCAGCTCGTGTCGTGAGAT
GTTGGGTTAAGTCCCGCAACGAGCGCAACCCTTGTCTCTAGTTGCTACGAAAGGGCACTCTAGAGAGACTGCCGGTGACAAACCGGAGGAAG
GTGGGGATGACGTCAAGTCCTCATGGCCCTTATGGGTAGGGCTTCACACGTCATACAATGGTGCATACAGAGGGTTGCCAAGCCGCGAGGTG
GAGCCAATCCCAGAAAATGCATCGTAGTCCGGATCGTAGTCTGCAACTCGACTACGTGAAGCTGGAATCGCTAGTAATCGCGGATCAGCATG
CCGCGGTGAATACGTTCCCGGGTCTTGTACACACCGCCCGTCACACCATGGGAGTGGGTTTTGCCAGAAGTAGTTAGCCTAACCGCAAGGAGG
GCGATTACCACGGCAGGGTTCATGACT
